# The relationship between fat distribution in central region and comorbidities in obese people: Based on NHANES 2011–2018

**DOI:** 10.3389/fendo.2023.1114963

**Published:** 2023-02-08

**Authors:** Chen-An Liu, Tong Liu, Guo-Tian Ruan, Yi-Zhong Ge, Meng-Meng Song, Hai-Lun Xie, Shi-Qi Lin, Li Deng, He-Yang Zhang, Qi Zhang, Han-Ping Shi

**Affiliations:** ^1^ Department of Gastrointestinal Surgery, Beijing Shijitan Hospital, Capital Medical University, Beijing, China; ^2^ Department of Clinical Nutrition, Beijing Shijitan Hospital, Capital Medical University, Beijing, China; ^3^ National Clinical Research Center for Geriatric Diseases, Xuanwu Hospital, Capital Medical University, Beijing, China; ^4^ Key Laboratory of Cancer Food for Special Medical Purposes (FSMP) for State Market Regulation, Beijing, China; ^5^ Beijing International Science and Technology Cooperation Base for Cancer Metabolism and Nutrition, Capital Medical University, Beijing, China; ^6^ Department of Colorectal Surgery, The Cancer Hospital of the University of Chinese Academy of Sciences, Zhejiang Cancer Hospital, Hangzhou, Zhejiang, China

**Keywords:** obesity, fat distribution, comorbidity, public health, NHANES

## Abstract

**Background:**

Central obesity is closely related to comorbidity, while the relationship between fat accumulation pattern and abnormal distribution in different parts of the central region of obese people and comorbidity is not clear. This study aimed to explore the relationship between fat distribution in central region and comorbidity among obese participants.

**Methods:**

We used observational data of NHANES 2011–2018 to identify 12 obesity-related comorbidities in 7 categories based on questionnaire responses from participants. Fat distribution is expressed by fat ratio, including Android, Gynoid, visceral, subcutaneous, visceral/subcutaneous (V/S), and total abdominal fat ratio. Logistic regression analysis were utilized to elucidate the association between fat distribution and comorbidity.

**Results:**

The comorbidity rate was about 54.1% among 4899 obese participants (weighted 60,180,984, 41.35 ± 11.16 years, 57.5% female). There were differences in fat distribution across the sexes and ages. Among men, Android fat ratio (OR, 4.21, 95% CI, 1.54–11.50, P_trend_=0.007), visceral fat ratio (OR, 2.16, 95% CI, 1.42–3.29, P_trend_<0.001) and V/S (OR, 2.07, 95% CI, 1.43–2.99, P_trend_<0.001) were independent risk factors for comorbidity. Among these, there was a “J” shape correlation between Android fat ratio and comorbidity risk, while visceral fat ratio and V/S exhibited linear relationships with comorbidity risk. The Gynoid fat ratio (OR, 0.87, 95%CI, 0.80–0.95, P_trend_=0.001) and subcutaneous fat ratio (OR, 0.81, 95%CI, 0.67–0.98, P_trend_=0.016) both performed a protective role in the risk of comorbidity. In women, Android fat ratio (OR, 4.65, 95% CI, 2.11–10.24, P_trend_=0.020), visceral fat ratio (OR, 1.83, 95% CI, 1.31–2.56, P_trend_=0.001), and V/S (OR, 1.80, 95% CI, 1.32–2.45, P_trend_=0.020) were also independent risk factors for comorbidity, with a dose-response relationship similar to that of men. Only the Gynoid fat ratio (OR, 0.93, 95% CI, 0.87–0.99, P_trend_=0.016) had a protective effect on female comorbidity. This association was also seen in obese participants of different age groups, comorbidity numbers, and comorbidity types, although it was more statistically significant in older, complex comorbidity, cardiovascular, cerebrovascular, and metabolic diseases.

**Conclusions:**

In the obese population, there were strong correlation between fat distribution in central region and comorbidity, which was affected by sex, age, number of comorbidities, and type of comorbidity.

## Introduction

Obesity is becoming more widespread all over the world, according to Global Burden of Disease Group research (1). Wang et al. predicted that by 2030, the total medical cost of obesity will double to 860.7-956.9 billion US dollars, accounting for about 16–18% of the total medical costs in the United States (2). It can threaten the health of people of any age, including induced cardiovascular disease (3), metabolic disease (4), liver disease (5), cancer (6), joint disease (7), and other comorbidities. However, these comorbidity studies focus mostly on the elderly and children, with little attention to adults aged 20–59 years, who account for the majority of the population distribution (8). This might be due to the low comorbidity rate of this age group. However, we must be aware that once young and middle-aged people are accompanied by these chronic diseases, they will be permanently and profoundly affected, and their quality of life and survival time may be significantly reduced. Therefore, we include this prevalent yet distinct category in this study.

Although obesity is closely related to a variety of comorbidities, the advent of the “obesity paradox” in recent years has broken everyone’s traditional understanding. Obesity based on body mass index (BMI) alone does not seem to well explain the protective effects of overweight and obesity in cardiovascular and cerebrovascular diseases (CCVD), cancer, and other diseases (9, 10), while individualized research on obesity types, body composition, lean and fat distribution have become increasingly valuable (11, 12).

We know that fat in obese people is often centrally accumulated, which is reflected in visceral fat, abdominal subcutaneous fat, hip fat and other regional fat. Previous studies have shown that the excessive distribution of Android fat and trunk fat may have a deleterious impact on subclinical right ventricular function, while the peripheral fat distribution may have a positive impact (13).

As far as we know, although some studies have conducted separate researches on waist hip ratio or visceral fat, no large-scale study has been conducted to explain the relationship between fat distribution in central region and comorbidities, even the number of comorbidities in obese patients of different ages and sexes. Therefore, this study aimed to explore the relationship between fat distribution and comorbidities such as CCVD, metabolic diseases (MD), respiratory diseases (RD), cancer, liver diseases, renal diseases, and joint diseases in obese adults aged 20-59 by analyzing the population in National Health and Nutrition Examination Survey (NHANES) database from 2011 to 2018, to provide us with a better scientific understanding of obesity and fat distribution in central region.

## Methods

### Participants and study design

The population of this study was sourced from the NHANES database—a large cross-sectional survey conducted by the National Center for Health Statistics—to investigate the health and nutritional status of the population in the United States (14–17). Its research design is complex and exquisite. The principal sample design consisted of multiyear, stratified, clustered 4-stage samples (18). According to the over-sampling standard, researchers over-sampled some subgroups of people and gave them corresponding weights so as to improve the accuracy and reliability of the overall data so that it can represent the demographic characteristics of the entire United States (19). On the official website of NHANES, we referred to the detailed survey contents, survey operations, and data-use methods (20). Personal information was mainly collected through personal interviews and mobile examination center, and all participants provided their signed informed consent (18).

In the present study, we analyzed 39,156 participants from the NHANES during 2011–2018, excluding the following patients: (1) participants aged <20 years, >59 years, and pregnant; (2) lack of data information that can be used to evaluate obesity (BMI and waist circumference); (3) non-obese participants; (4) lack of fat mass data; (5) lack of baseline data (such as income, marital status, smoking, and drinking); (6) lack of comorbidity information. Finally, we included 4,899 obese participants (60,180,984 participants after weighting). The screening process is depicted in **Figure S1**.

### Exposure variables and definitions

In this study, all participants were examined by dual-energy X-ray absorptiometry (DXA) to determine the fat mass, which is the most widely accepted method of measuring body composition (21). The fat distribution in the central region includes Android, Gynoid, visceral, subcutaneous, visceral/subcutaneous (V/S), and total abdominal fat ratio (See the **Supplementary materials** for the definition of these areas). The fat distribution was described by the ratio (%), that is, the fat mass of each part/total fat mass × 100%. Obesity was defined as BMI ≥30 or waist circumference (wc) ≥88 cm in women or wc ≥102 cm in men (22).

### Outcome

Our primary study outcome was the comorbidity risk among obese participants. We obtained whether the patient also had other diseases from the medical conditions file in the NHANES questionnaire section. We included 12 obesity-related diseases in 7 categories reported previously, which included CCVD (such as hypertension, coronary heart disease, heart failure, and stroke), MD (diabetes and gout), RD (asthma, chronic bronchitis), liver disease, renal disease, and cancer and joint diseases. Among these, simple comorbidity is defined as <4 comorbidities, while the participants with ≥4 diseases were defined as complex comorbidities (23).

### Statistical analyses

Considered the complex survey design of NHANES, all statistical analysis was based on sample weight, stratification, and clustering. Continuous variables were expressed by means ± standard deviation (SD), and categorical variables were expressed by percentage (%). Continuous variables were compared with the Student’s *t*-test or non-parametric test, and the categorical variables were compared with the Rao-Scott Chi-square test. Considering the large difference in the distribution of fat between men and women, we classified the study participants into 2 groups of men and women and applied logistic regression analysis to clarify the relationship between the distribution of fat in different portions and the risk of comorbidity. For continuous variables that did not conform to the normal distribution, we conducted a natural logarithm transformation and also described these variables in the form of sex-specific quintiles. The cutoff value was calculated from the ROC curve. In addition, we used the variance inflation factor (VIF) to detect multicollinearity among covariates. VIF >10 was considered to indicate multicollinearity.

In order to clarify the correlation between fat distribution and comorbidity risk among different subgroups, we analyzed the age subgroups (<45 and ≥45 years), comorbidity number subgroups (simple comorbidity and complex comorbidity), and comorbidity-type subgroups. In order to test the robustness of the results, we performed a sensitivity analysis, adjusted the age subgroups to <40 and ≥ 40 years old, and then performed a logistic regression analysis to clarify the relationship between fat distribution and comorbidity. Two-sided P < 0.05 was considered to indicate statistical significance, and all statistical analyses were performed by the R software (Version 4.1.2).

## Result

### Characteristics of study participants

The mean age (SD) of these 4899 obese participants was 41.35 ± 11.16 years. The majority of them were female, with about 2950 participants. We found significant differences in total fat mass (32.84 ± 8.94 kg vs 36.56 ± 10.52 kg) and fat distribution between men and women (P < 0.001), including Android, Gynoid, visceral, subcutaneous, and abdominal fat ratio and V/S (**Table 1**).

**Table 1:** Clinical characteristics and body measurements of study participants in NHANES 2011-2018.

	Male (n=1950)	Female (n=2949)	P value
Weighted sample size, No. (%)	25,553,771 (42.5)	34,627,213 (57.5)	
Age (mean)	41.37 (11.12)	41.34 (11.19)	0.941
Race, No. (%)			<0.001
Mexican American	358 (12.7)	528 (11.0)	
Other Hispanic	214 (7.9)	340 (7.3)	
Non-Hispanic White	760 (63.2)	1043 (61.9)	
Non-Hispanic Black	387 (9.4)	717 (13.1)	
Other Race	231 (6.8)	321 (6.7)	
Education, No. (%)			<0.001
Less than high school	359 (12.4)	533 (12.5)	
High school or equivalent	523 (27.2)	617 (20.4)	
College or above	1068 (60.3)	1799 (67.1)	
Marital, No. (%)			<0.001
Married	1091 (58.4)	1413 (53.1)	
Separated	218 (11.6)	586 (18.3)	
Never married	641 (30.0)	950 (28.7)	
Ratio of family income to poverty, No. (%)			<0.001
0-1.0	445 (15.1)	848 (21.2)	
1.1-3.0	806 (36.0)	1161 (35.1)	
>3.0	699 (48.9)	940 (43.7)	
Medical insurance, No. (%)			0.019
No	543 (21.3)	680 (17.8)	
Yes	1407 (78.7)	2269 (82.2)	
Alcohol drinking, No. (%)			<0.001
No	312 (13.9)	954 (24.6)	
Yes	1638 (86.1)	1995 (75.4)	
Smoke, No. (%)			<0.001
No	1014 (52.6)	1927 (61.4)	
Yes	936 (47.4)	1022 (38.6)	
BMI (mean)	33.29 (4.70)	32.48 (6.41)	<0.001
Arm circumference (mean)	37.59 (3.54)	34.72 (4.56)	<0.001
Waist (mean)	112.32 (10.94)	104.73 (13.36)	<0.001
Total fat mass (kg, mean)	32.84 (8.94)	36.56 (10.52)	<0.001
Android fat ratio (mean)	10.34 (1.27)	8.52 (1.31)	<0.001
Gynoid fat ratio (mean)	15.48 (1.76)	17.11 (2.11)	<0.001
Visceral fat ratio (mean)	2.22 (0.76)	1.65 (0.60)	<0.001
Subcutaneous fat ratio (mean)	5.69 (0.66)	6.48 (0.83)	<0.001
Visceral to Subcutaneous fat ratio (mean)	0.40 (0.15)	0.26 (0.10)	<0.001
Abdominal fat ratio (mean)	8.06 (0.91)	8.27 (1.06)	<0.001
Comorbidity, No. (%)	1074 (53.6)	1640 (54.4)	0.626

The majority of patients had comorbidity (54.1%), and there was no significant difference in the comorbidity rates between men and women (P = 0.626). The comorbidity rate of men was about 53.6%, while women had about 54.4%. The comorbidity types of the two groups were similar, including hypertension (men, 32.6%, women, 26.5%), arthritis (men, 14.9%, women, 20.9%) and asthma (men, 12.8%, women, 19%).

### Relationship between fat distribution and comorbidity risk in different sexes

After adjusting for age, race, education level, marital status, income, medical insurance, alcohol drinking, smoking, BMI, wc, and arm circumference, the Android fat ratio (OR, 4.21, 95% CI, 1.54–11.50, P_trend_=0.007), visceral fat ratio (OR, 2.16, 95% CI, 1.42–3.29, P_trend_<0.001), and V/S (OR, 2.07, 95% CI, 1.43–2.99, P_trend_<0.001) were independent risk factors for comorbidity in men. The Android fat ratio is “J” type related to comorbidity risk, while the visceral fat ratio and V/S were very significantly linear type related to comorbidity risk, that is, compared to Quintile 1, the OR values of Quintiles 2, 3, 4, and 5 exhibited progressive growth. Simultaneously, the Gynoid fat ratio (OR, 0.87, 95%CI, 0.80–0.95, P_trend_=0.001) and the subcutaneous fat ratio (OR, 0.81, 95%CI, 0.67–0.98, P_trend_=0.016) played a protective role in the risk of comorbidity, and this trend was still visible after dividing by the cutoff value. After dividing by sex-specific quintiles, we discovered a significant linear inversely dose-response relationship between Gynoid fat ratio, subcutaneous fat ratio, and comorbidity risk, implying that their protective effects were accumulated as fat ratio increases (**Table 2**).

**Table 2:** Odds ratio (95%CI) of comorbidity risk with different fat distribution in male obese people.

		Android fat(%)		Gynoid fat(%)		Visceral fat(%)	
		OR (95%CI)	P-value	OR (95%CI)	P-value	OR (95%CI)	P-value
As continuous (per SD)		4.21 (1.54, 11.50)	0.006	0.87 (0.80, 0.95)	0.002	2.16 (1.42, 3.29)	0.001
By cut-off	Low	Ref		Ref		Ref	
	High	1.58 (1.20, 2.05)	0.001	0.46 (0.33,0.62)	<0.001	1.67 (1.20,2.31)	0.003
Quintile	Q1	Ref		Ref		Ref	
	Q2	0.75 (0.50, 1.12)	0.154	0.44 (0.30, 0.64)	<0.001	1.39 (0.92, 2.10)	0.112
	Q3	0.97 (0.69, 1.36)	0.856	0.33 (0.21, 0.50)	<0.001	1.62 (1.02, 2.57)	0.042
	Q4	1.05 (0.69, 1.62)	0.805	0.37 (0.25, 0.56)	<0.001	2.05 (1.31, 3.19)	0.002
	Q5	1.53 (1.01, 2.33)	0.047	0.32 (0.20, 0.50)	<0.001	2.35 (1.44, 3.84)	0.001
	Ptrend		0.007		0.001		<0.001
		Subcutaneous fat(%)		V/S		Abdominal fat(%)	
As continuous (per SD)		0.81 (0.67, 0.98)	0.032	2.07 (1.43, 2.99)	<0.001	1.13 (0.96,1.32)	0.13
By cut-off	Low	Ref		Ref		Ref	
	High	0.60 (0.47,0.77)	<0.001	2.10 (1.62,2.72)	<0.001	1.37 (1.05,1.79)	0.022
Quintile	Q1	Ref		Ref		Ref	
	Q2	0.89 (0.61, 1.31)	0.553	1.53 (1.12, 2.10)	0.008	0.70 (0.49, 1.02)	0.062
	Q3	0.80 (0.55, 1.17)	0.249	2.04 (1.38, 3.02)	0.001	0.90 (0.58, 1.40)	0.634
	Q4	0.61 (0.39, 0.96)	0.033	2.42 (1.66, 3.54)	<0.001	1.06 (0.68, 1.67)	0.779
	Q5	0.66 (0.42, 1.05)	0.078	3.92 (2.54, 6.07)	<0.001	1.00 (0.61, 1.64)	0.996
	Ptrend		0.016		<0.001		0.298

Adjusted for Age, Race, Education, Marital status, Ratio of family income to poverty, Medical insurance, Smoke, Alcohol, BMI, Waist, Arm circumference.

We also adjusted for covariates among women. The results showed that as continuous variables, Android fat ratio (OR, 4.65, 95% CI, 2.11–10.24, P_trend_=0.020), visceral fat ratio (OR, 1.83, 95% CI, 1.31–2.56, P_trend_=0.001), and V/S (OR, 1.80, 95% CI, 1.32–2.45, P_trend_=0.020) were also independent risk factors for comorbidity. And their dose-response association showed the same trend as in males. Unlike the male results, however, only the Gynoid fat ratio (OR, 0.93, 95% CI, 0.87–0.99, P_trend_=0.016) played a protective role (**Table 3**). Although there was a protective trend in the subcutaneous fat ratio (OR, 0.69, 95% CI, 0.31–1.53), it was not statistically significant (P = 0.350, P_trend_=0.208).

**Table 3:** Odds ratio (95%CI) of comorbidity risk with different fat distribution in female obese people.

		Android fat (%)		Gynoid fat (%)		Visceral fat(%)	
		OR (95%CI)	P-value	OR (95%CI)	P-value	OR (95%CI)	P-value
As continuous (per SD)		4.65 (2.11, 10.24)	<0.001	0.93 (0.87, 0.99)	0.022	1.83 (1.31, 2.56)	0.001
By cut-off	Low	Ref		Ref		Ref	
	High	1.37 (1.09, 1.73)	0.009	0.64 (0.49, 0.83)	0.001	1.71 (1.31, 2.22)	<0.001
Quintile	Q1	Ref		Ref		Ref	
	Q2	0.91 (0.65, 1.28)	0.586	0.57 (0.39, 0.83)	0.004	1.08 (0.79, 1.50)	0.617
	Q3	1.16 (0.82, 1.65)	0.401	0.46 (0.32, 0.66)	<0.001	1.03 (0.77, 1.38)	0.815
	Q4	1.11 (0.77, 1.60)	0.565	0.46 (0.32, 0.67)	<0.001	1.41 (1.01, 1.97)	0.044
	Q5	1.55 (1.04, 2.31)	0.030	0.55 (0.37, 0.82)	0.004	2.18 (1.35, 3.50)	0.002
	P_trend_		0.020		0.016		0.001
		Subcutaneous fat(%)		V/S		Abdominal fat(%)	
As continuous (per SD)		0.69 (0.31, 1.53)	0.35	1.80 (1.32, 2.45)	<0.001	1.04 (0.94, 1.16)	0.398
By cut-off	Low	Ref		Ref		Ref	
	High	0.85 (0.68, 1.06)	0.136	2.13 (1.67, 2.74)	<0.001	1.26 (0.99, 1.60)	0.061
Quintile	Q1	Ref		Ref		Ref	
	Q2	0.81 (0.59, 1.10)	0.169	1.23 (0.91, 1.67)	0.173	0.87 (0.66, 1.16)	0.331
	Q3	0.92 (0.66, 1.28)	0.605	1.36 (1.03, 1.79)	0.032	1.01 (0.75, 1.38)	0.926
	Q4	0.72 (0.52, 0.98)	0.036	1.72 (1.20, 2.46)	0.004	1.05 (0.76, 1.45)	0.755
	Q5	0.92 (0.68, 1.25)	0.578	3.55 (2.30, 5.49)	<0.001	1.06 (0.76, 1.46)	0.739
	P_trend_		0.208		<0.001		0.371

Adjusted for Age, Race, Education, Marital status, Ratio of family income to poverty, Medical insurance, Smoke, Alcohol, BMI, Waist, Arm circumference.

### Relationship between fat distribution and comorbidity risk stratified by age

**Figure 1:**
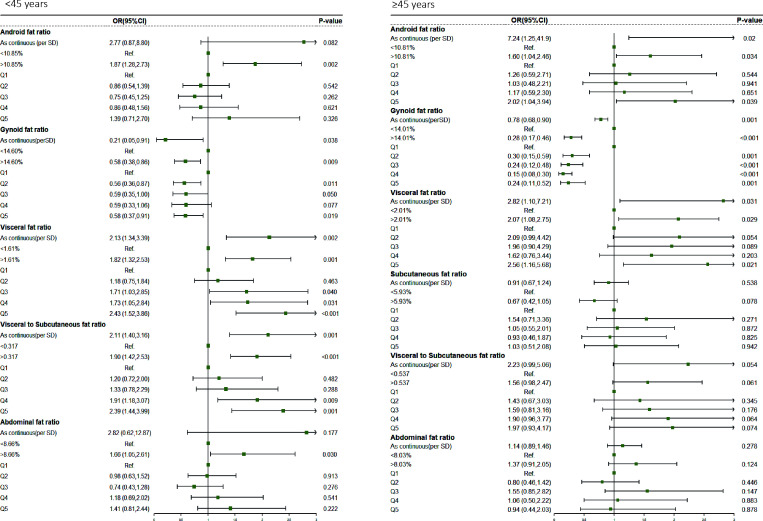
Comorbidity risk odds ratio (95% CI) of different fat distribution in obese male.

**Figure 2:**
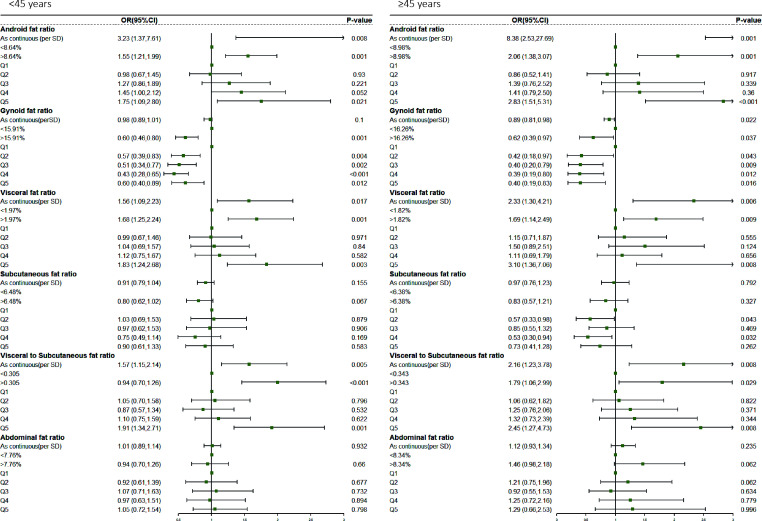
Comorbidity risk odds ratio (95% CI) of different fat distribution in obese female.

We separated men and women into two groups(<45 and≥45 years old) to explore the differences in fat distribution and comorbidity across age groups. We found variations in all fat ratio among participants of two groups, regardless of sex (**Figure S2**). Further logistic regression analysis showed that (**Tables S1, S2**; **Figure 1**), in contrast to the results of the total male population, the Android fat ratio (OR, 2.77, 95%CI, 0.87–8.80, P = 0.082) and the Gynoid fat ratio (OR, 0.91, 95%CI, 0.83–1.00, P = 0.056) of men with <45 were not statistically significant with the risk of comorbidity. But in men with ≥45, Android fat ratio (OR, 7.24, 95%CI, 1.25-41.49, P = 0.020) and Gynoid fat ratio (OR, 0.78, 95%CI, 0.68–0.90, P = 0.001) were significantly associated with comorbidity risk, and this trend also existed in women (**Tables S3, S4**; **Figure 2**)

### Relationship between fat distribution and risk of complex comorbidity

**Figure 3:**
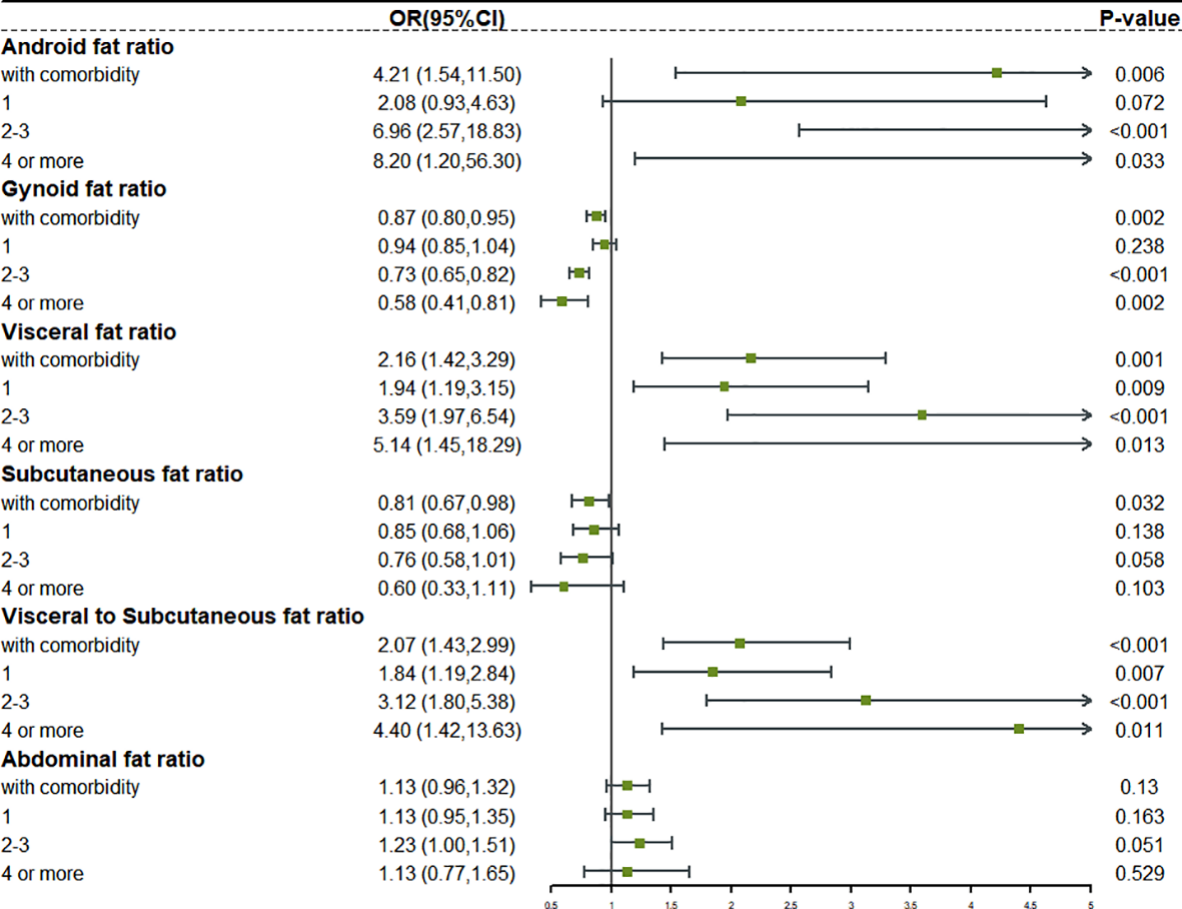
The relationship between different fat distribution and different degrees of comorbidity in obese male.

**Figure 4:**
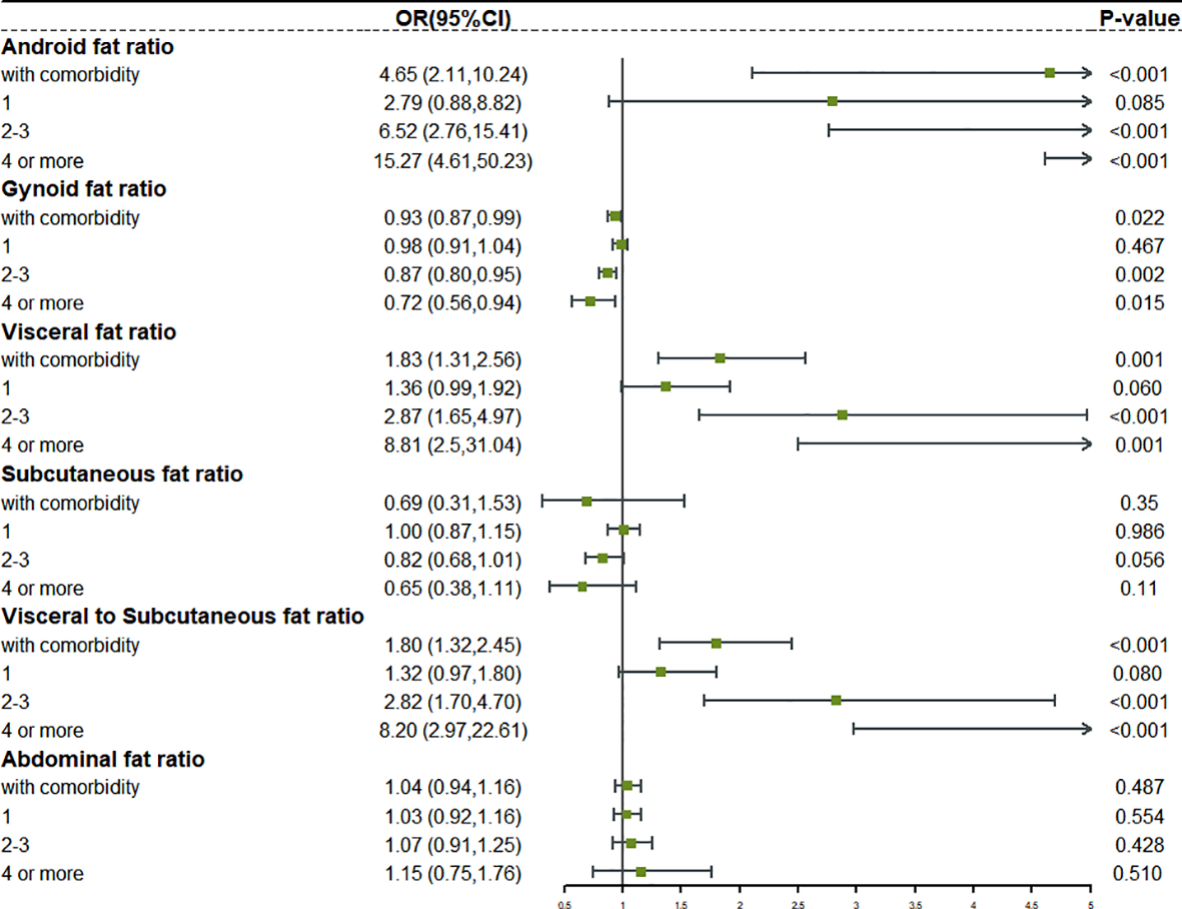
The relationship between different fat distribution and different degrees of comorbidity in obese female.

We reclassified the patients according to the number of comorbidities. Complex comorbidities were defined as four or more comorbidities. Based on this, we studied the relationship between fat distribution as a continuous variable and various degrees of comorbidity (**Figures S3, S4**). The forest plot in **Figures 3**, **4** clearly showed that, with the emergence of complex comorbidity, Android fat ratio, visceral fat ratio, and V/S have significantly increased the risk of comorbidity, while the protective effect of Gynoid fat ratio and the subcutaneous fat ratio on the risk of comorbidity had also increased.

### Relationship between fat distribution and risk of different types of comorbidities

We reclassified the patients according to their comorbidity types, mainly including CCVD, MD, RD, liver disease, renal disease, cancer, and joint disease, and studied the relationship between fat distribution and different types of comorbidities. As continuous and categorical variables, the quintile OR value of CCVD comorbidity risk of Android fat ratio, visceral fat ratio, and V/S showed a significant increase. Similarly, the quintiles OR value of Gynoid fat ratio and subcutaneous fat ratio, and CCVD comorbidity risk showed a decreasing trend, indicating that its protective effect was gradually increasing (**Figure S5**). Except for subcutaneous fat ratio, Android fat ratio, Gynoid fat ratio, visceral fat ratio, and V/S were the same in MD as they were in CCVD (**Figure S6**). However, when fat distribution was associated with the risk of RD, liver disease, renal disease, cancer, and joint disease, these relationships were less regular (**Figures S7–S11**).

### Sensitive analysis

In addition, we also conducted some sensitivity analyses and discovered that the results were stable. To overcome the bias caused by age grouping, we reset the age boundary and investigated the role of fat distribution in obese participants at the age of 40. Android fat ratio (OR, 2.60, 95% CI, 0.56–12.12, P = 0.219) and Gynoid fat ratio (OR, 0.96, 95% CI, 0.86–1.08, P = 0.501) were not significantly associated with comorbidity among male obese adults aged <40 years (**Figure S12**). Conversely, the risk effect of Android fat ratio (OR, 5.30, 95% CI, 1.33–21.13, P = 0.019) and the protective effect of Gynoid fat ratio (OR, 0.80, 95% CI, 0.71–0.91, P = 0.006) were obvious for male obese people aged ≥40 years. In women, the results were similar (**Figure S13**). Considering the impact of menstrual status on female fat distribution and some disease risks (CCVD), the results of subgroups of people with menstrual status by age showed that the comorbidity risk of postmenopausal (older) participants seemed more likely to be affected by abnormal fat distribution (**Figure S14**). In addition, in order to avoid the impact of estrogen use, we made additional adjustments to the estrogen use of participants with complete estrogen use information, and the results were still stable (**Table S5**).

## Discussion

We analyzed 60 million obese individuals aged 20–59 years in this large-scale prospective study that can represent the majority of the US population. The results showed that even the central regional fat was highly heterogeneous, with different fat distributions having distinct consequences on comorbidity risk. In obese patients, the Android fat ratio, visceral fat ratio, and V/S were all independent risk factors for comorbidity. The Gynoid fat ratio accumulation provided protection. Furthermore, in men, the accumulation of abdominal subcutaneous fat performed a protective role in the risk of comorbidity. However, the change in total abdominal fat had no discernable effect on the incidence of comorbidity. Further subgroup analysis showed that the effects of fat distribution were more strongly correlated with comorbidity risk in older participants, as well as complex comorbidity, CCVD, and MD.

### Fat distribution and clinical characteristics

This study initially investigated the differences in fat distribution among obese participants of different sexes and ages. To begin, males had greater Android fat, visceral fat, and V/S compared to women, but less Gynoid fat and subcutaneous fat, which may be related to hormone levels, eating habits, living habits, and genetic differences (24). Second, this difference was mirrored in fat function. We also discovered that in men, both visceral fat ratio and subcutaneous fat ratio were strongly linked with comorbidity risk, but in women, only visceral fat ratio was significantly associated with comorbidity risk. This result was completely consistent with the results of Mutsert et al. (25). As a result, in women, just variations in visceral fat may need to be assessed for stratification of comorbidity risk, but in men, the potential effects of subcutaneous fat may need to be additionally assessed. Second, age was an important reason for the differences in fat distribution among participants. With advancing age, Android fat, visceral fat, and abdominal fat increased, but Gynoid fat and subcutaneous fat decreased. This also coincided with previous research results. Aging promotes fat redistribution, that is, loss of subcutaneous fat and growth of visceral fat, and hormonal imbalance can also invert the distribution of Android and Gynoid fat (26). In terms of fat function, older participants were more susceptible to fat than younger participants, which was consistent with previous studies. Preis et al. also found a stronger correlation between fat distribution and metabolic diseases in older participants (27).

### Fat distribution and complex comorbidity

For obese participants, complex comorbidities are a difficult public health prevention target (28). Although some studies have noted the relationship between fat distribution or obesity degree and various comorbidities, for example, a recent study by Mika et al. found a dose-response relationship between obese individuals’ BMI and complex comorbidities, obese participants exhibited a 5-fold greater risk of simple comorbidity and a 12-fold increased risk of complex comorbidity compared to healthy weight participants (23). However, few studies have elucidated the relationship between fat distribution and complex comorbidity. When compared to people with simple comorbidity, the fat distribution of participants with complex comorbidity was more closely related to comorbidity risk, and this trend was not affected by sex. The results of this study were unprecedented because it effectively filled the deficiency in previous studies that relied solely on BMI to determine the risk of complex comorbidity.

### Fat distribution and comorbidity type

A large number of studies have shown a strong correlation between obesity and various types of comorbidities, with the most widely reported comorbidities being cardiovascular, metabolic, and respiratory diseases (29–31). Albert et al. showed that obesity can cause a variety of hemodynamic changes, which may lead to cardiac morphological changes and ventricular dysfunction (32). Although this theory has been widely confirmed, we cannot ignore the latest research on the obesity paradox in cardiovascular disease. The mortality of patients with any kind of heart failure has decreased as BMI has increased (33). This contradictory phenomenon prompts us to focus our research on body composition. We found that increasing the Android fat ratio, visceral fat ratio, and V/S would increase the risk of CCVD and even the mortality of special causes. However, the Gynoid fat ratio and subcutaneous fat ratio played considerable protective roles. This conclusion has not been explored in depth before and it may be a reasonable explanation for the “obesity paradox”. The increase in BMI will not benefit all obese people. Participants will not benefit from a rise in BMI induced by Android and visceral fat. Only the increase in BMI caused by Gynoid and subcutaneous fat may achieve the effect of the “obesity paradox”. Similarly, while obesity is associated with the occurrence of MD such as diabetes and gout (34, 35), the risk of MD caused by fat in different regions was not the same.

The most reasonable explanation for this phenomenon is fat heterogeneity. The Android fat and visceral fat are composed of white adipose tissues (WAT), which contribute to metabolism and chronic inflammation *in vivo*, while triglycerides accumulation in WAT cells in obese people triggers WAT cells remodeling, proliferation, and hypertrophy. The ERK and p38 MAPK pathways are activated by adipocytokines secretion, resulting in increased CCL2 expression in adipocytes. This in turn triggers pro-inflammatory macrophage aggregation, Treg cell reduction, and IL-6 and TNF-α secretion increases, leading to systemic inflammation, insulin resistance, oxidative stress, and a series of metabolic reactions (36, 37). As for Gynoid fat, estrogen induction increases the anti-lipolytic α2-adrenergic receptors in the gluteal-femoral subcutaneous fat depot, causing fat to accumulate in the Gynoid area; hence, Gynoid fat distribution is closely related to estrogen levels (38). Estrogen has been widely recognized as an important factor in regulating obesity and metabolic balance in the body (39). Tran’s study showed that estrogen-regulated multiple calcium-dependent activities in cardiovascular tissues *via* influencing calcium signaling mechanism components (40). Alternatively, by activating eNOS and increasing NO production, as well as activating cardioprotective signaling cascades including Akt and MAP kinases, cardiac and endothelial cells are protected against apoptosis and necrosis, alleviating pathological myocardial hypertrophy (41). Estrogen also plays an important role in metabolic pathways. Animal experiments have shown that estrogen can increase insulin content and glucose-stimulated insulin secretion in isolated mouse islets, and maintain glucose homeostasis, while its deficiency will disturb oxidative stress and endoplasmic reticulum function, resulting in a complete disorder of insulin function and *in vivo* metabolism. Therefore, the accumulation of Gynoid fat caused by increased estrogen significantly reduces the risk of comorbidity in obese people.

Interestingly, the increase in total abdominal fat did not appear to affect the risk of any type of comorbidity in this study, which differs slightly from previous reports indicating total abdominal fat was an independent risk factor for cardiovascular disease (42). Visceral fat is primarily responsible for the risk of total abdominal fat, while subcutaneous fat has a protective effect. Therefore, we specially analyzed the role of V/S in comorbidity. It has been proved that V/S can be used to assess the risk of comorbidity, but merely judging total abdominal fat cannot be very effective in guiding clinical work. Individual evaluation of visceral fat, abdominal subcutaneous fat and V/S is necessary to meet the requirements of precision nutrition and precision medicine.

## Strengths and limitations

Obesity has been on the rise in adults since the 1980s, but over the past decade, the prevalence of obesity and severe obesity has continued to increase among young and middle-aged adults aged 20 to 59 years, compared to those aged <20 years and >60 years (43), despite previous studies focusing primarily on adolescents and the elderly. This is the first study to systematically study the fat distribution and comorbidity risk, complex comorbidity, and comorbidity types of obese people aged 20–59 years. Second, the part of our study on complex comorbidities is of great public health significance. Obese individuals aged <50 years had a higher risk of complex comorbidity than older obese participants, and the extremely high complex comorbidity rate imposes a huge socioeconomic burden (23). This study’s population is obese people aged 20–59 years, so it may effectively guide public health prevention and control and reduce the complex comorbidity rate of such people, improving their quality of life and survival time. Notwithstanding, our study also had some limitations. First, since this is a cross-sectional study, we could not obtain the dynamic changes in the participants’ body composition, which may lead to unclear causality; second, we did not account for diet, exercise, and lifestyle habits, which could confound our results. Finally, changes in menstrual status, hormone treatment and hormone level may affect the distribution and mass of fat, thus affecting the results. However, due to the limitations of NHANES database, we did not adjust these covariants. In future clinical research, we will pay more attention to these aspects.

## Conclusions

Taken together, these results have clinical and public health implications, and our study highlights the correlation between fat distribution and comorbidity, which is influenced by sex, age, number of comorbidities, and type of comorbidity. As we age, we should pay more attention to changes in central fat distribution, and people with abnormal fat distribution should be on the lookout for CCVD and MD. Furthermore, because of the strong correlation between abnormal fat distribution and complex comorbidities, it is particularly important to distinguish the fat function of various parts of obese people. This result provides clinical guidance that obesity treatment (such as life intervention, pharmacotherapy and bariatric surgery) should be used with greater caution and precision for young and middle-aged obese people.

## Data availability statement

The original contributions presented in the study are included in the article/**Supplementary material**. Further inquiries can be directed to the corresponding author.

## Ethics statement

The studies involving human participants were reviewed and approved by National Center for Health Statistics. The patients/participants provided their written informed consent to participate in this study.

## Author contributions

Conceptualization, H-PS and C-AL; methodology, G-TR, H-LX; software, S-QL, Y-ZG and C-AL; validation, M-MS, TL, and C-AL; investigation, H-PS; resources, C-AL; data curation, C-AL and S-QL; writing—original draft preparation, C-AL; writing—review and editing, C-AL, M-MS, G-TR, LD and H-LX; visualization, QZ, and TL; supervision, C-AL and H-PS; project administration, H-PS. All authors contributed to the article and approved the submitted version.
