# Supplementary material for: The relationship between fat distribution in central region and comorbidities in obese people: Based on NHANES 2011–2018
Source: Front Endocrinol (Lausanne). 2023 Feb 8;14:1114963. doi: 10.3389/fendo.2023.1114963 (PMC9945539; doi:10.3389/fendo.2023.1114963)

Table S1 Comorbidity risk odds ratio (95%CI) of different fat distribution in obese men younger than 45 years old

|  | As continuous (per SD) |  |  |  | By cut-off |  |  |  |  | Quintile |  |  |  |  |
| --- | --- | --- | --- | --- | --- | --- | --- | --- | --- | --- | --- | --- | --- | --- |
|  | OR | Lower | Upper | P-value |  | OR | Lower | Upper | P-value |  | OR | Lower | Upper | P-value |
| Android fat（%） | 2.77 | 0.87 | 8.80 | 0.082 | ≤10.85% | Ref |  |  |  | Q1 | Ref |  |  |  |
|  |  |  |  |  | ＞10.85% | 1.87 | 1.28 | 2.73 | 0.002 | Q2 | 0.86 | 0.54 | 1.39 | 0.542 |
|  |  |  |  |  |  |  |  |  |  | Q3 | 0.75 | 0.45 | 1.25 | 0.262 |
|  |  |  |  |  |  |  |  |  |  | Q4 | 0.86 | 0.48 | 1.56 | 0.621 |
|  |  |  |  |  |  |  |  |  |  | Q5 | 1.39 | 0.71 | 2.70 | 0.326 |
| Gynoid fat（%） | 0.91 | 0.83 | 1.00 | 0.056 | ≤14.60% | Ref |  |  |  | Q1 | Ref |  |  |  |
|  |  |  |  |  | ＞14.60% | 0.58 | 0.38 | 0.86 | 0.009 | Q2 | 0.56 | 0.36 | 0.87 | 0.011 |
|  |  |  |  |  |  |  |  |  |  | Q3 | 0.59 | 0.35 | 1.00 | 0.05 |
|  |  |  |  |  |  |  |  |  |  | Q4 | 0.59 | 0.33 | 1.06 | 0.077 |
|  |  |  |  |  |  |  |  |  |  | Q5 | 0.58 | 0.37 | 0.91 | 0.019 |
| Visceral fat（%） | 2.13 | 1.34 | 3.39 | 0.002 | ≤1.61% | Ref |  |  |  | Q1 | Ref |  |  |  |
|  |  |  |  |  | ＞1.61% | 1.82 | 1.32 | 2.53 | 0.001 | Q2 | 0.94 | 0.55 | 1.63 | 0.825 |
|  |  |  |  |  |  |  |  |  |  | Q3 | 1.52 | 0.85 | 2.72 | 0.157 |
|  |  |  |  |  |  |  |  |  |  | Q4 | 1.53 | 0.88 | 2.65 | 0.125 |
|  |  |  |  |  |  |  |  |  |  | Q5 | 1.86 | 1.05 | 3.31 | 0.034 |
| Subcutaneous fat（%） | 0.77 | 0.61 | 0.97 | 0.029 | ≤5.96% | Ref |  |  |  | Q1 | Ref |  |  |  |
|  |  |  |  |  | ＞5.96% | 0.70 | 0.51 | 0.96 | 0.026 | Q2 | 0.64 | 0.40 | 1.03 | 0.063 |
|  |  |  |  |  |  |  |  |  |  | Q3 | 0.70 | 0.45 | 1.09 | 0.112 |
|  |  |  |  |  |  |  |  |  |  | Q4 | 0.55 | 0.32 | 0.94 | 0.029 |
|  |  |  |  |  |  |  |  |  |  | Q5 | 0.59 | 0.37 | 0.93 | 0.024 |
| V/S | 2.11 | 1.40 | 3.16 | 0.001 | <0.317 | Ref |  |  |  | Q1 | Ref |  |  |  |
|  |  |  |  |  | >0.317 | 1.90 | 1.42 | 2.53 | ＜0.001 | Q2 | 1.20 | 0.72 | 2.00 | 0.482 |
|  |  |  |  |  |  |  |  |  |  | Q3 | 1.33 | 0.78 | 2.29 | 0.288 |
|  |  |  |  |  |  |  |  |  |  | Q4 | 1.91 | 1.18 | 3.07 | 0.009 |
|  |  |  |  |  |  |  |  |  |  | Q5 | 2.39 | 1.44 | 3.99 | 0.001 |
| Abdominal fat（%） | 1.16 | 0.96 | 1.41 | 0.130 | ≤8.66% | Ref |  |  |  | Q1 | Ref |  |  |  |
|  |  |  |  |  | ＞8.66% | 1.66 | 1.05 | 2.61 | 0.03 | Q2 | 0.98 | 0.63 | 1.52 | 0.913 |
|  |  |  |  |  |  |  |  |  |  | Q3 | 0.74 | 0.43 | 1.28 | 0.276 |
|  |  |  |  |  |  |  |  |  |  | Q4 | 1.18 | 0.69 | 2.02 | 0.541 |
|  |  |  |  |  |  |  |  |  |  | Q5 | 1.41 | 0.81 | 2.44 | 0.222 |

Adjusted for Race, Education, Marital status, Ratio of family income to poverty, Medical insurance, Smoke, Alcohol, BMI, Waist, Arm circumference.

Table S2 Comorbidity risk odds ratio (95%CI) of different fat distribution in obese men aged 45 years or older

|  | As continuous (per SD) |  |  |  | By cut-off |  |  |  |  | Quintile |  |  |  |  |
| --- | --- | --- | --- | --- | --- | --- | --- | --- | --- | --- | --- | --- | --- | --- |
|  | OR | Lower | Upper | P-value |  | OR | Lower | Upper | P-value |  | OR | Lower | Upper | P-value |
| Android fat（%） | 7.24 | 1.25 | 41.90 | 0.020 | ≤10.81% | Ref |  |  |  | Q1 | Ref |  |  |  |
|  |  |  |  |  | ＞10.81% | 1.60 | 1.04 | 2.46 | 0.034 | Q2 | 1.26 | 0.59 | 2.71 | 0.544 |
|  |  |  |  |  |  |  |  |  |  | Q3 | 1.03 | 0.48 | 2.21 | 0.941 |
|  |  |  |  |  |  |  |  |  |  | Q4 | 1.17 | 0.59 | 2.30 | 0.651 |
|  |  |  |  |  |  |  |  |  |  | Q5 | 2.02 | 1.04 | 3.94 | 0.039 |
| Gynoid fat（%） | 0.78 | 0.68 | 0.90 | 0.001 | ≤14.01% | Ref |  |  |  | Q1 | Ref |  |  |  |
|  |  |  |  |  | ＞14.01% | 0.28 | 0.17 | 0.46 | ＜0.001 | Q2 | 0.30 | 0.15 | 0.59 | 0.001 |
|  |  |  |  |  |  |  |  |  |  | Q3 | 0.24 | 0.12 | 0.48 | ＜0.001 |
|  |  |  |  |  |  |  |  |  |  | Q4 | 0.15 | 0.08 | 0.30 | ＜0.001 |
|  |  |  |  |  |  |  |  |  |  | Q5 | 0.24 | 0.11 | 0.52 | 0.001 |
| Visceral fat（%） | 2.82 | 1.10 | 7.21 | 0.031 | ≤2.01% | Ref |  |  |  | Q1 | Ref |  |  |  |
|  |  |  |  |  | ＞2.01% | 2.07 | 1.08 | 2.75 | 0.029 | Q2 | 2.09 | 0.99 | 4.42 | 0.054 |
|  |  |  |  |  |  |  |  |  |  | Q3 | 1.96 | 0.90 | 4.29 | 0.089 |
|  |  |  |  |  |  |  |  |  |  | Q4 | 1.62 | 0.76 | 3.44 | 0.203 |
|  |  |  |  |  |  |  |  |  |  | Q5 | 2.56 | 1.16 | 5.68 | 0.021 |
| Subcutaneous fat（%） | 0.91 | 0.67 | 1.24 | 0.538 | ≤5.93% | Ref |  |  |  | Q1 | Ref |  |  |  |
|  |  |  |  |  | ＞5.93% | 0.67 | 0.42 | 1.05 | 0.078 | Q2 | 1.54 | 0.71 | 3.36 | 0.271 |
|  |  |  |  |  |  |  |  |  |  | Q3 | 1.05 | 0.55 | 2.01 | 0.872 |
|  |  |  |  |  |  |  |  |  |  | Q4 | 0.93 | 0.46 | 1.87 | 0.825 |
|  |  |  |  |  |  |  |  |  |  | Q5 | 1.03 | 0.51 | 2.08 | 0.942 |
| V/S | 2.23 | 0.99 | 5.06 | 0.054 | ≤0.537 |  |  |  |  | Q1 | Ref |  |  |  |
|  |  |  |  |  | >0.537 | 1.56 | 0.98 | 2.47 | 0.061 | Q2 | 1.43 | 0.67 | 3.03 | 0.345 |
|  |  |  |  |  |  |  |  |  |  | Q3 | 1.59 | 0.81 | 3.16 | 0.176 |
|  |  |  |  |  |  |  |  |  |  | Q4 | 1.90 | 0.96 | 3.77 | 0.064 |
|  |  |  |  |  |  |  |  |  |  | Q5 | 1.97 | 0.93 | 4.17 | 0.074 |
| Abdominal fat（%） | 1.14 | 0.89 | 1.46 | 0.278 | ≤8.03% | Ref |  |  |  | Q1 | Ref |  |  |  |
|  |  |  |  |  | ＞8.03% | 1.37 | 0.91 | 2.05 | 0.124 | Q2 | 0.8 | 0.46 | 1.42 | 0.446 |
|  |  |  |  |  |  |  |  |  |  | Q3 | 1.55 | 0.85 | 2.82 | 0.147 |
|  |  |  |  |  |  |  |  |  |  | Q4 | 1.06 | 0.50 | 2.22 | 0.883 |
|  |  |  |  |  |  |  |  |  |  | Q5 | 0.94 | 0.44 | 2.03 | 0.878 |

Adjusted for Race, Education, Marital status, Ratio of family income to poverty, Medical insurance, Smoke, Alcohol, BMI, Waist, Arm circumference.

Table S3 Comorbidity risk odds ratio (95%CI) of different fat distribution in obese femen younger than 45 years old

|  | As continuous (per SD) |  |  |  | By cut-off |  |  |  |  | Quintile |  |  |  |  |
| --- | --- | --- | --- | --- | --- | --- | --- | --- | --- | --- | --- | --- | --- | --- |
|  | OR | Lower | Upper | P-value |  | OR | Lower | Upper | P-value |  | OR | Lower | Upper | P-value |
| Android fat（%） | 3.23 | 1.37 | 7.61 | 0.008 | ≤8.64% | Ref |  |  |  | Q1 | Ref |  |  |  |
|  |  |  |  |  | ＞8.64% | 1.55 | 1.21 | 1.99 | 0.001 | Q2 | 0.98 | 0.67 | 1.45 | 0.93 |
|  |  |  |  |  |  |  |  |  |  | Q3 | 1.27 | 0.86 | 1.89 | 0.221 |
|  |  |  |  |  |  |  |  |  |  | Q4 | 1.45 | 1.00 | 2.12 | 0.052 |
|  |  |  |  |  |  |  |  |  |  | Q5 | 1.75 | 1.09 | 2.8 | 0.021 |
| Gynoid fat（%） | 0.98 | 0.89 | 1.01 | 0.100 | ≤15.91% | Ref |  |  |  | Q1 | Ref |  |  |  |
|  |  |  |  |  | ＞15.91% | 0.60 | 0.46 | 0.80 | 0.001 | Q2 | 0.57 | 0.39 | 0.83 | 0.004 |
|  |  |  |  |  |  |  |  |  |  | Q3 | 0.51 | 0.34 | 0.77 | 0.002 |
|  |  |  |  |  |  |  |  |  |  | Q4 | 0.43 | 0.28 | 0.65 | <0.001 |
|  |  |  |  |  |  |  |  |  |  | Q5 | 0.6 | 0.4 | 0.89 | 0.012 |
| Visceral fat（%） | 1.56 | 1.09 | 2.23 | 0.017 | ≤1.97% | Ref |  |  |  | Q1 | Ref |  |  |  |
|  |  |  |  |  | ＞1.97% | 1.68 | 1.25 | 2.24 | 0.001 | Q2 | 0.99 | 0.67 | 1.46 | 0.971 |
|  |  |  |  |  |  |  |  |  |  | Q3 | 1.04 | 0.69 | 1.57 | 0.84 |
|  |  |  |  |  |  |  |  |  |  | Q4 | 1.12 | 0.75 | 1.67 | 0.582 |
|  |  |  |  |  |  |  |  |  |  | Q5 | 1.83 | 1.24 | 2.68 | 0.003 |
| Subcutaneous fat（%） | 0.91 | 0.79 | 1.04 | 0.155 | ≤6.48% | Ref |  |  |  | Q1 | Ref |  |  |  |
|  |  |  |  |  | ＞6.48% | 0.80 | 0.62 | 1.02 | 0.067 | Q2 | 1.03 | 0.69 | 1.53 | 0.879 |
|  |  |  |  |  |  |  |  |  |  | Q3 | 0.97 | 0.62 | 1.53 | 0.906 |
|  |  |  |  |  |  |  |  |  |  | Q4 | 0.75 | 0.49 | 1.14 | 0.169 |
|  |  |  |  |  |  |  |  |  |  | Q5 | 0.9 | 0.61 | 1.33 | 0.583 |
| V/S | 1.57 | 1.15 | 2.14 | 0.005 | ≤0.305 | Ref |  |  |  | Q1 | Ref |  |  |  |
|  |  |  |  |  | >0.305 | 2.00 | 1.46 | 2.73 | <0.001 | Q2 | 1.05 | 0.70 | 1.58 | 0.796 |
|  |  |  |  |  |  |  |  |  |  | Q3 | 0.87 | 0.57 | 1.34 | 0.532 |
|  |  |  |  |  |  |  |  |  |  | Q4 | 1.10 | 0.75 | 1.59 | 0.622 |
|  |  |  |  |  |  |  |  |  |  | Q5 | 1.91 | 1.34 | 2.71 | 0.001 |
| Abdominal fat（%） | 1.01 | 0.89 | 1.14 | 0.932 | ≤7.76% | Ref |  |  |  | Q1 | Ref |  |  |  |
|  |  |  |  |  | ＞7.76% | 0.94 | 0.7 | 1.26 | 0.660 | Q2 | 0.92 | 0.61 | 1.39 | 0.677 |
|  |  |  |  |  |  |  |  |  |  | Q3 | 1.07 | 0.71 | 1.63 | 0.732 |
|  |  |  |  |  |  |  |  |  |  | Q4 | 0.97 | 0.63 | 1.51 | 0.894 |
|  |  |  |  |  |  |  |  |  |  | Q5 | 1.05 | 0.72 | 1.54 | 0.798 |

Adjusted for Race, Education, Marital status, Ratio of family income to poverty, Medical insurance, Smoke, Alcohol, BMI, Waist, Arm circumference.

Table S4 Comorbidity risk odds ratio (95%CI) of different fat distribution in obese femen aged 45 years or older

|  | As continuous (per SD) |  |  |  | By cut-off |  |  |  |  | Quintile |  |  |  |  |
| --- | --- | --- | --- | --- | --- | --- | --- | --- | --- | --- | --- | --- | --- | --- |
|  | OR | Lower | Upper | P-value |  | OR | Lower | Upper | P-value |  | OR | Lower | Upper | P-value |
| Android fat（%） | 7.95 | 2.22 | 28.42 | 0.002 | ≤8.98% | Ref |  |  |  | Q1 | Ref |  |  |  |
|  |  |  |  |  | ＞8.98% | 2.02 | 1.33 | 3.06 | 0.001 | Q2 | 0.85 | 0.53 | 1.38 | 0.507 |
|  |  |  |  |  |  |  |  |  |  | Q3 | 1.34 | 0.73 | 2.49 | 0.339 |
|  |  |  |  |  |  |  |  |  |  | Q4 | 1.32 | 0.72 | 2.41 | 0.36 |
|  |  |  |  |  |  |  |  |  |  | Q5 | 2.76 | 1.44 | 5.27 | 0.003 |
| Gynoid fat（%） | 0.89 | 0.81 | 0.98 | 0.022 | ≤16.26% | Ref |  |  |  | Q1 | Ref |  |  |  |
|  |  |  |  |  | ＞16.26% | 0.62 | 0.39 | 0.97 | 0.037 | Q2 | 0.42 | 0.18 | 0.97 | 0.043 |
|  |  |  |  |  |  |  |  |  |  | Q3 | 0.40 | 0.20 | 0.79 | 0.009 |
|  |  |  |  |  |  |  |  |  |  | Q4 | 0.39 | 0.19 | 0.80 | 0.012 |
|  |  |  |  |  |  |  |  |  |  | Q5 | 0.4 | 0.19 | 0.83 | 0.016 |
| Visceral fat（%） | 2.33 | 1.30 | 4.21 | 0.006 | ≤1.82% | Ref |  |  |  | Q1 | Ref |  |  |  |
|  |  |  |  |  | ＞1.82% | 1.69 | 1.14 | 2.49 | 0.009 | Q2 | 1.15 | 0.71 | 1.87 | 0.555 |
|  |  |  |  |  |  |  |  |  |  | Q3 | 1.50 | 0.89 | 2.51 | 0.124 |
|  |  |  |  |  |  |  |  |  |  | Q4 | 1.11 | 0.69 | 1.79 | 0.656 |
|  |  |  |  |  |  |  |  |  |  | Q5 | 3.10 | 1.36 | 7.06 | 0.008 |
| Subcutaneous fat（%） | 0.97 | 0.76 | 1.23 | 0.792 | ≤6.38% | Ref |  |  |  | Q1 | Ref |  |  |  |
|  |  |  |  |  | ＞6.38% | 0.83 | 0.57 | 1.21 | 0.327 | Q2 | 0.57 | 0.33 | 0.98 | 0.043 |
|  |  |  |  |  |  |  |  |  |  | Q3 | 0.85 | 0.55 | 1.32 | 0.469 |
|  |  |  |  |  |  |  |  |  |  | Q4 | 0.53 | 0.30 | 0.94 | 0.032 |
|  |  |  |  |  |  |  |  |  |  | Q5 | 0.73 | 0.41 | 1.28 | 0.262 |
| V/S | 2.16 | 1.23 | 3.78 | 0.008 | ≤0.343 | Ref |  |  |  | Q1 | Ref |  |  |  |
|  |  |  |  |  | >0.343 | 1.79 | 1.06 | 2.99 | 0.029 | Q2 | 1.06 | 0.62 | 1.82 | 0.822 |
|  |  |  |  |  |  |  |  |  |  | Q3 | 1.32 | 0.73 | 2.39 | 0.344 |
|  |  |  |  |  |  |  |  |  |  | Q4 | 1.25 | 0.76 | 2.06 | 0.371 |
|  |  |  |  |  |  |  |  |  |  | Q5 | 2.45 | 1.27 | 4.73 | 0.008 |
| Abdominal fat（%） | 1.12 | 0.93 | 1.34 | 0.235 | ≤8.34% | Ref |  |  |  | Q1 | Ref |  |  |  |
|  |  |  |  |  | ＞8.34% | 1.46 | 0.98 | 2.18 | 0.062 | Q2 | 1.21 | 0.75 | 1.96 | 0.425 |
|  |  |  |  |  |  |  |  |  |  | Q3 | 0.92 | 0.55 | 1.53 | 0.749 |
|  |  |  |  |  |  |  |  |  |  | Q4 | 1.25 | 0.72 | 2.16 | 0.421 |
|  |  |  |  |  |  |  |  |  |  | Q5 | 1.29 | 0.66 | 2.53 | 0.448 |

Adjusted for Race, Education, Marital status, Ratio of family income to poverty, Medical insurance, Smoke, Alcohol, BMI, Waist, Arm circumference.

Table S5 Odds ratio (95%CI) of comorbidity risk with different fat distribution in participants with complete estrogen treatment information

|  | As continuous (per SD) |  |  | As continuous (per SD) |  |
| --- | --- | --- | --- | --- | --- |
|  | OR (95%CI) | P-value |  | OR (95%CI) | P-value |
| Android fat（%） | 1.15 (1.01, 1.31) | 0.037 | Subcutaneous fat（%） | 1 (0.90, 1.11) | 0.960 |
| Gynoid fat（%） | 0.85 (0.75, 0.96) | 0.013 | V/S | 1.30 (1.11, 1.52) | 0.002 |
| Visceral fat（%） | 1.31 (1.12, 1.52) | 0.001 | Abdominal fat（%） | 1.08 (0.97, 1.21) | 0.140 |

Adjusted for Age, Race, Education, Marital status, Ratio of family income to poverty, Medical insurance, Smoke, Alcohol, BMI, Waist, Arm circumference and estrogen use.

Figure S1 Flow chart of the participants in this study


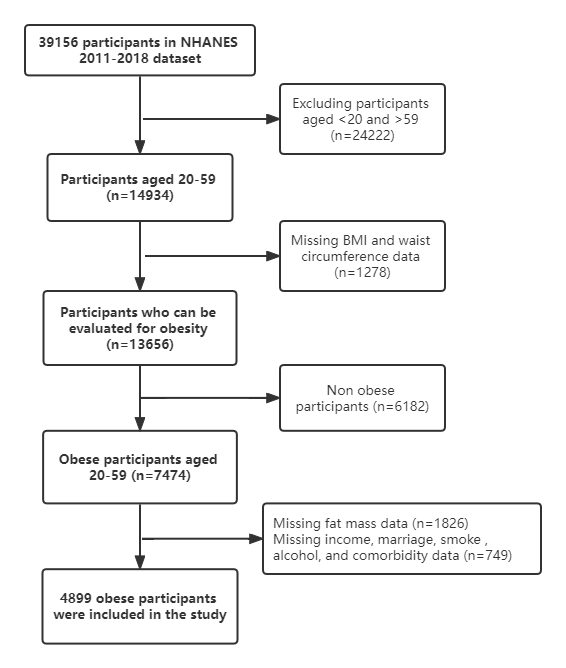


Figure S2 Differences in fat distribution among obese people of different ages (< 45 years old and ≥ 45 years old)


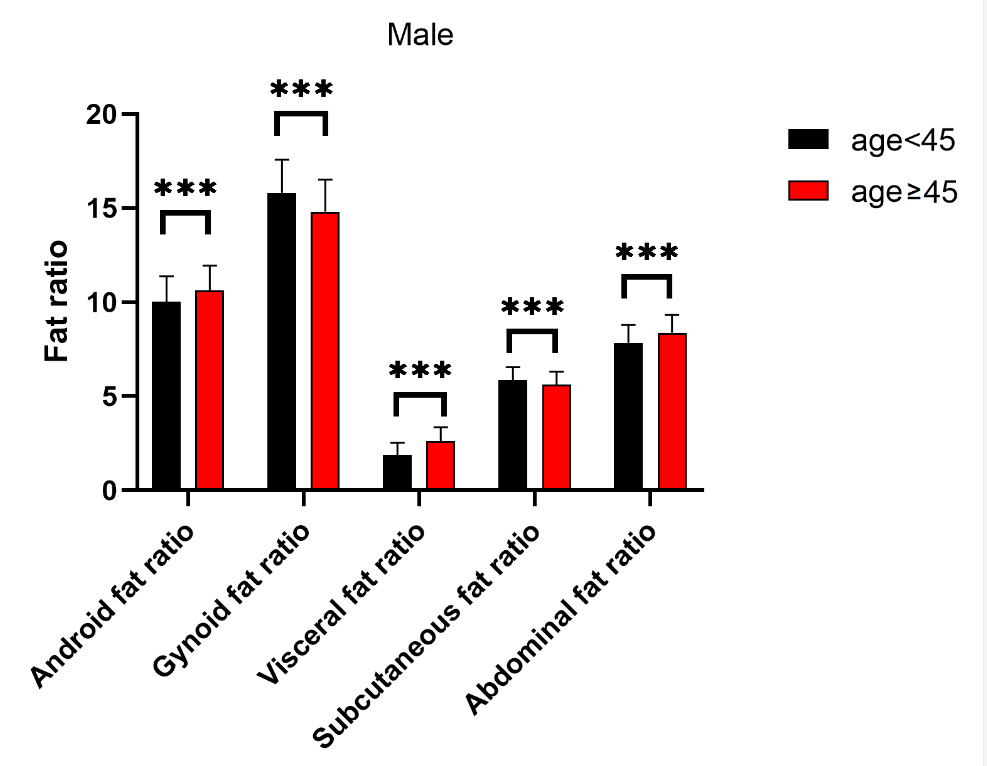

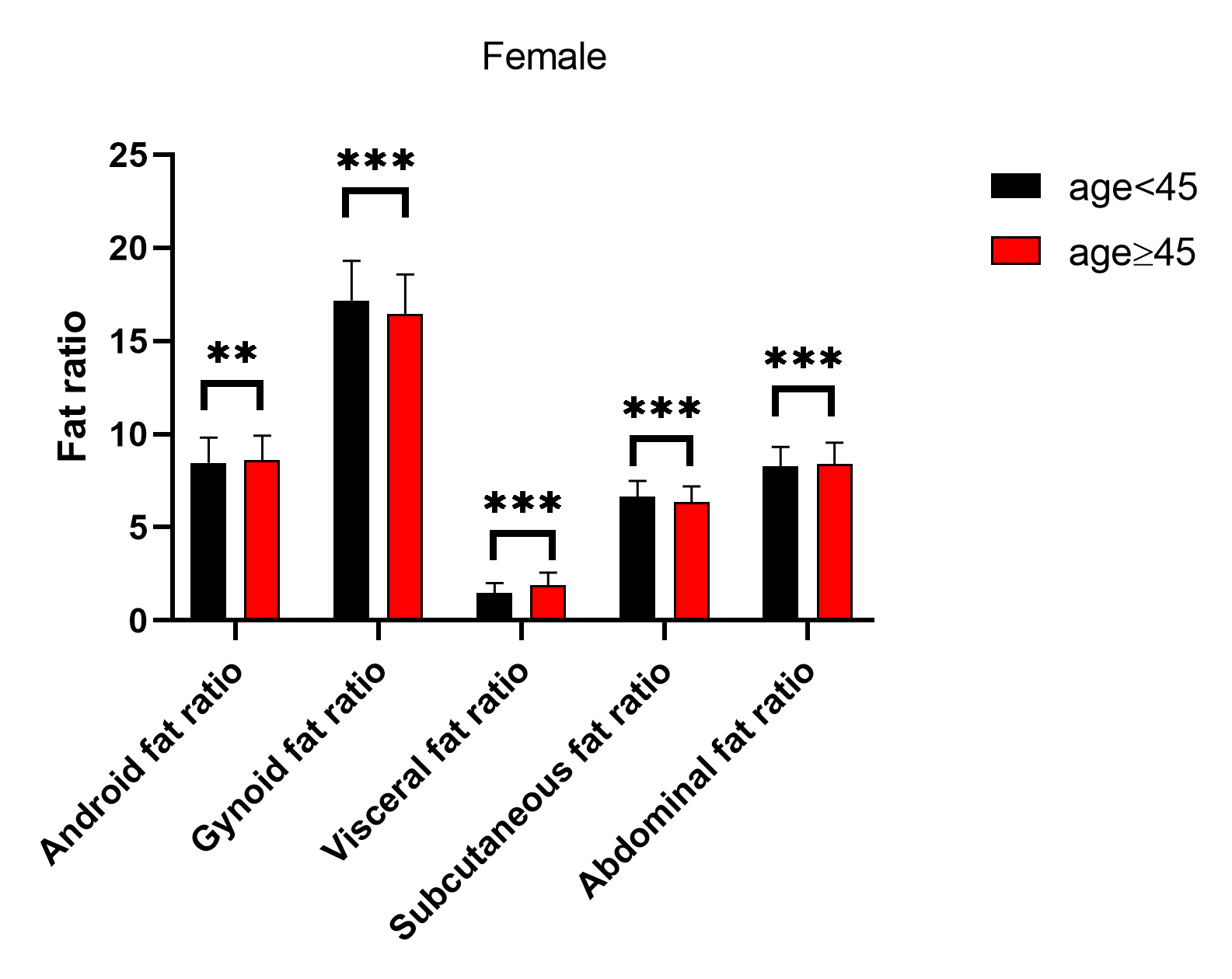


Figure S3 Correlation analysis between fat ratio and number of comorbidities in men participants of different ages (0: < 45 years old, 1: ≥ 45 years old)


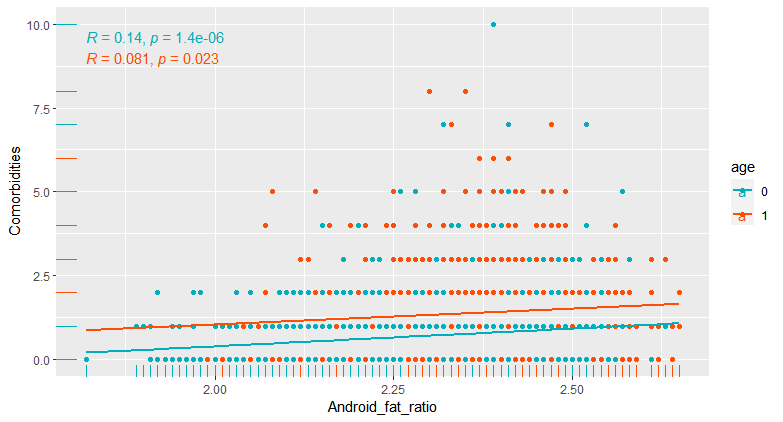

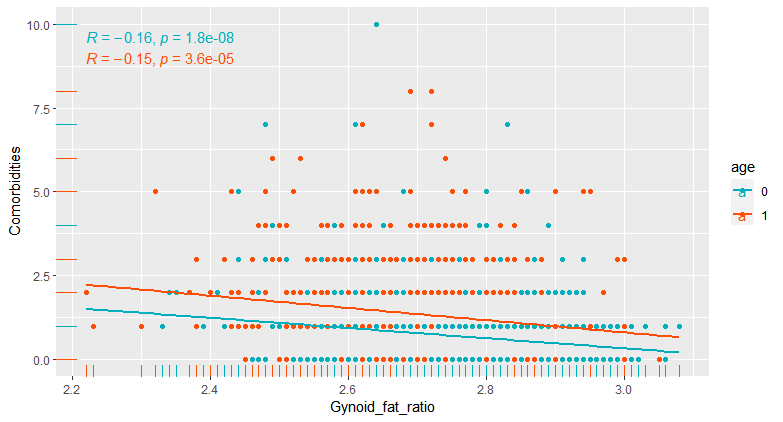

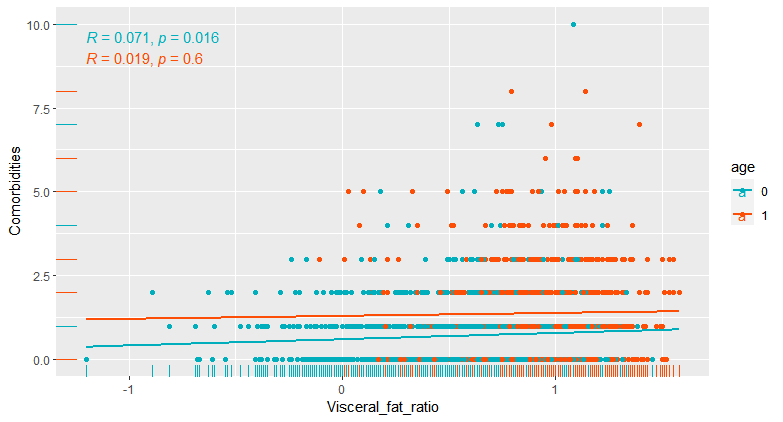

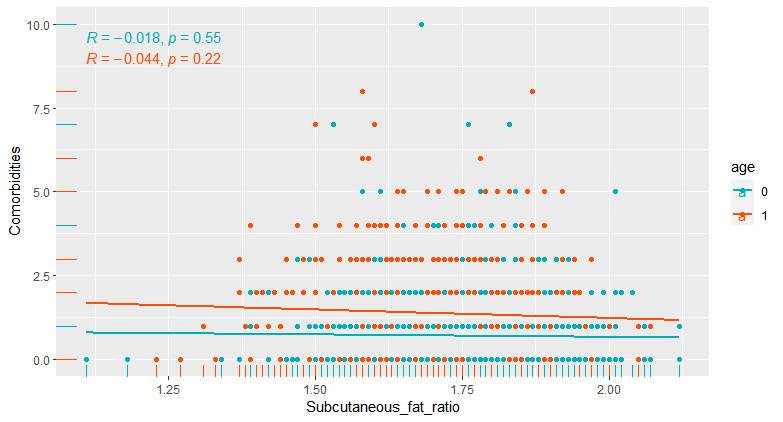


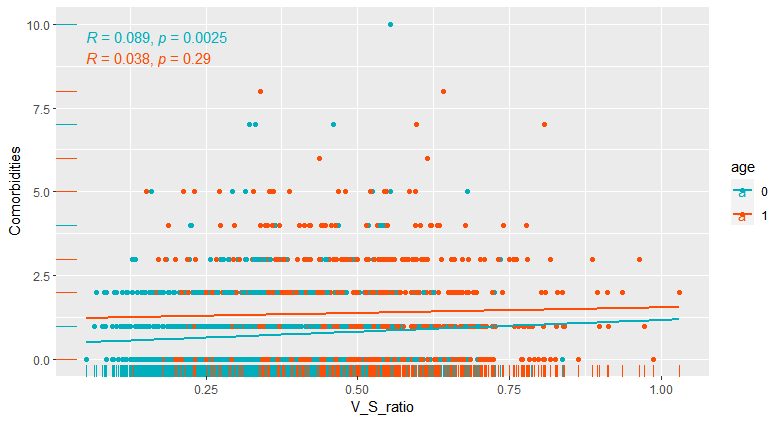

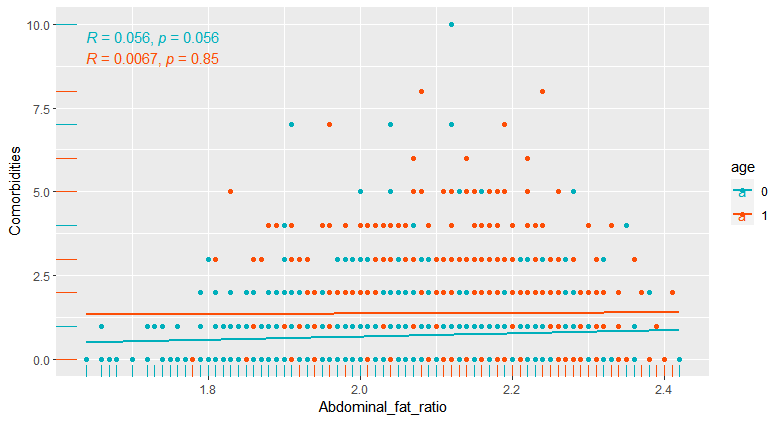


Figure S4 Correlation analysis between fat ratio and number of comorbidities in femen participants of different ages (0: < 45 years old, 1: ≥ 45 years old)


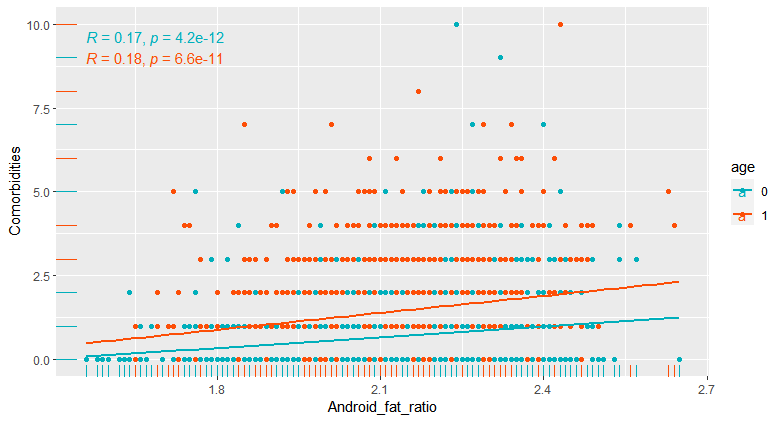

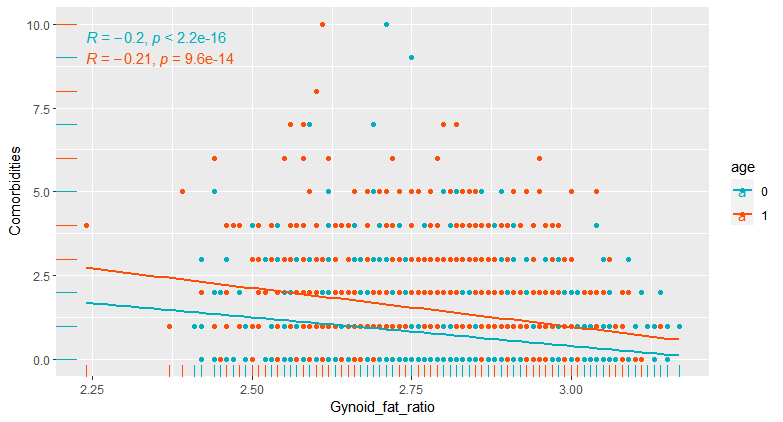

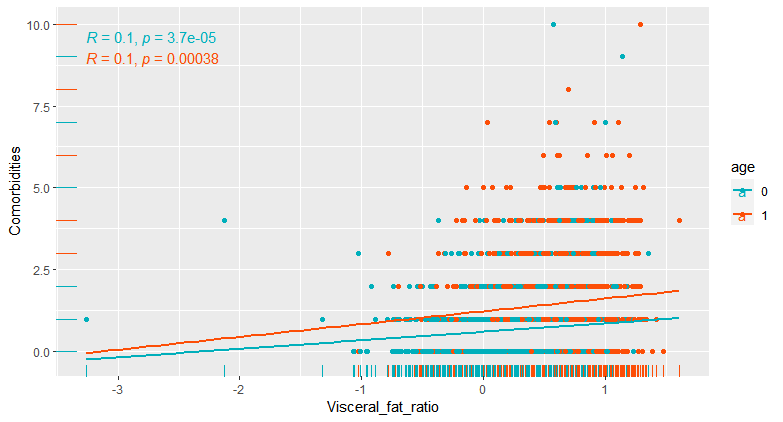

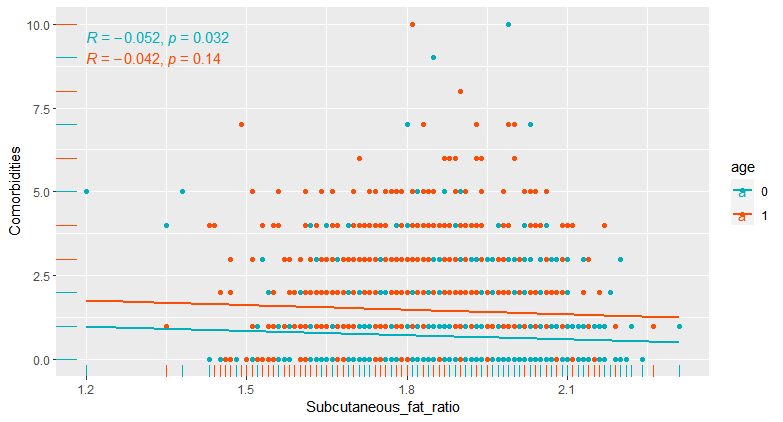

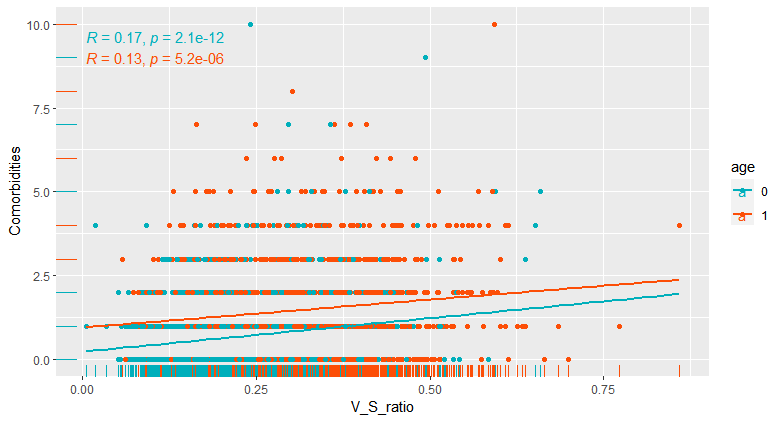

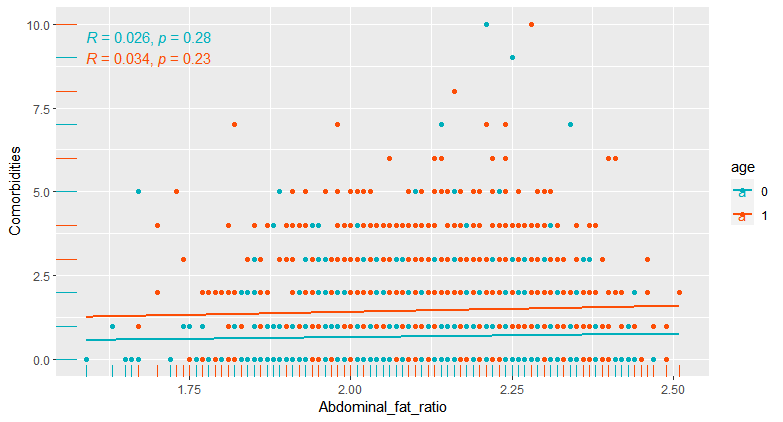


Figure S5 Relationship between different fat distribution and CCVD in obese participants


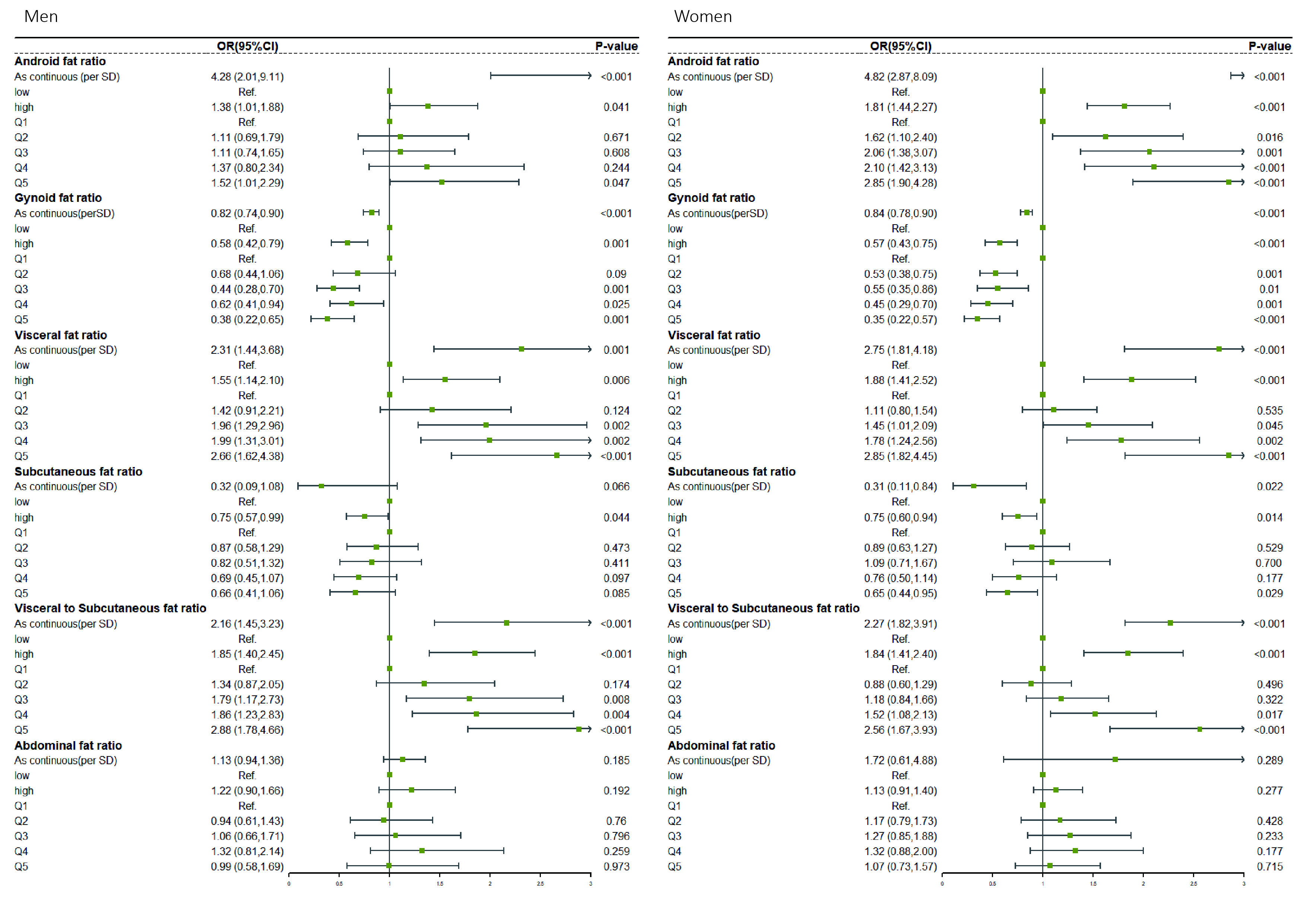


Figure S6 Relationship between different fat distribution and MD in obese participants


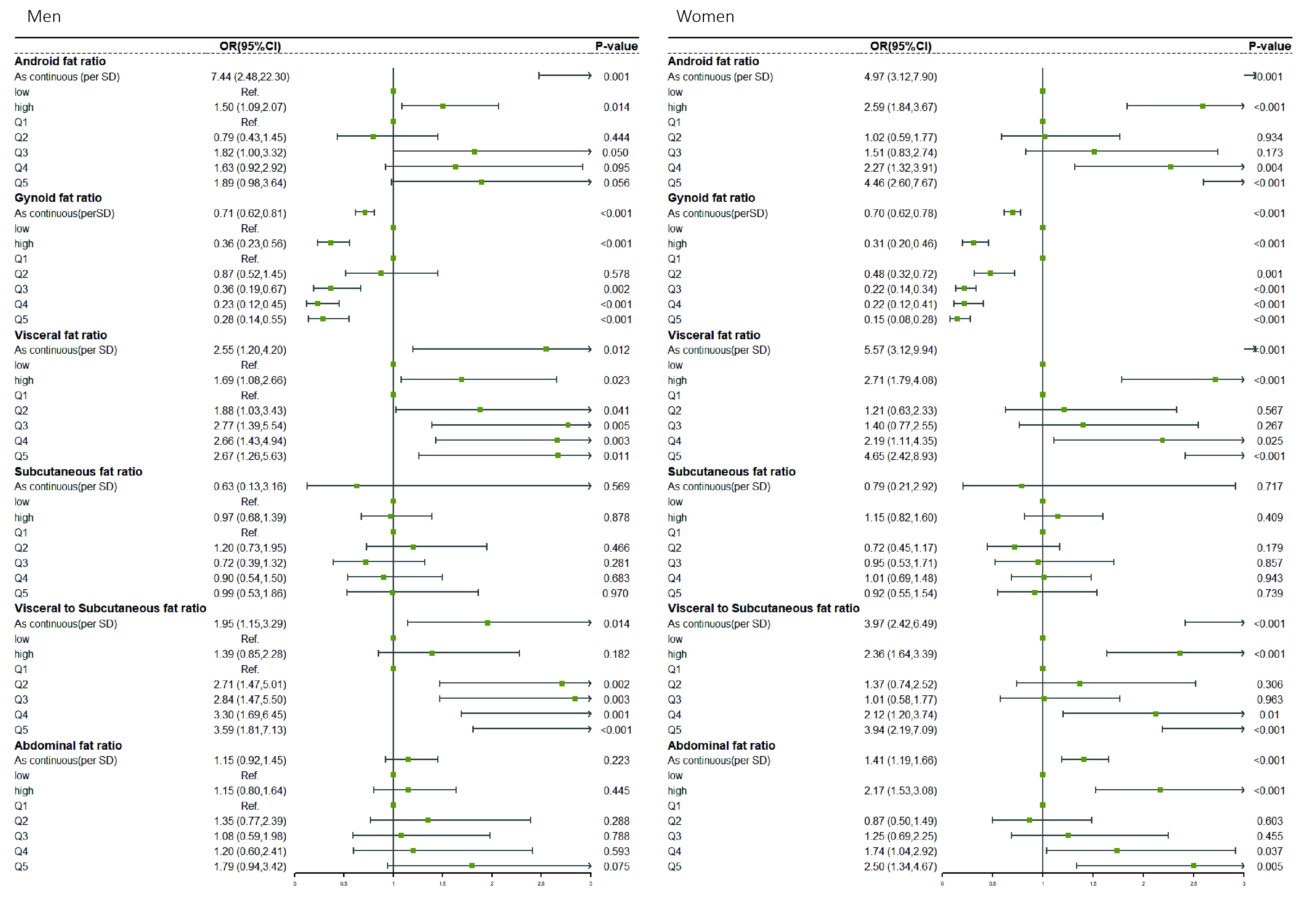


Figure S7 Relationship between different fat distribution and RD in obese participants


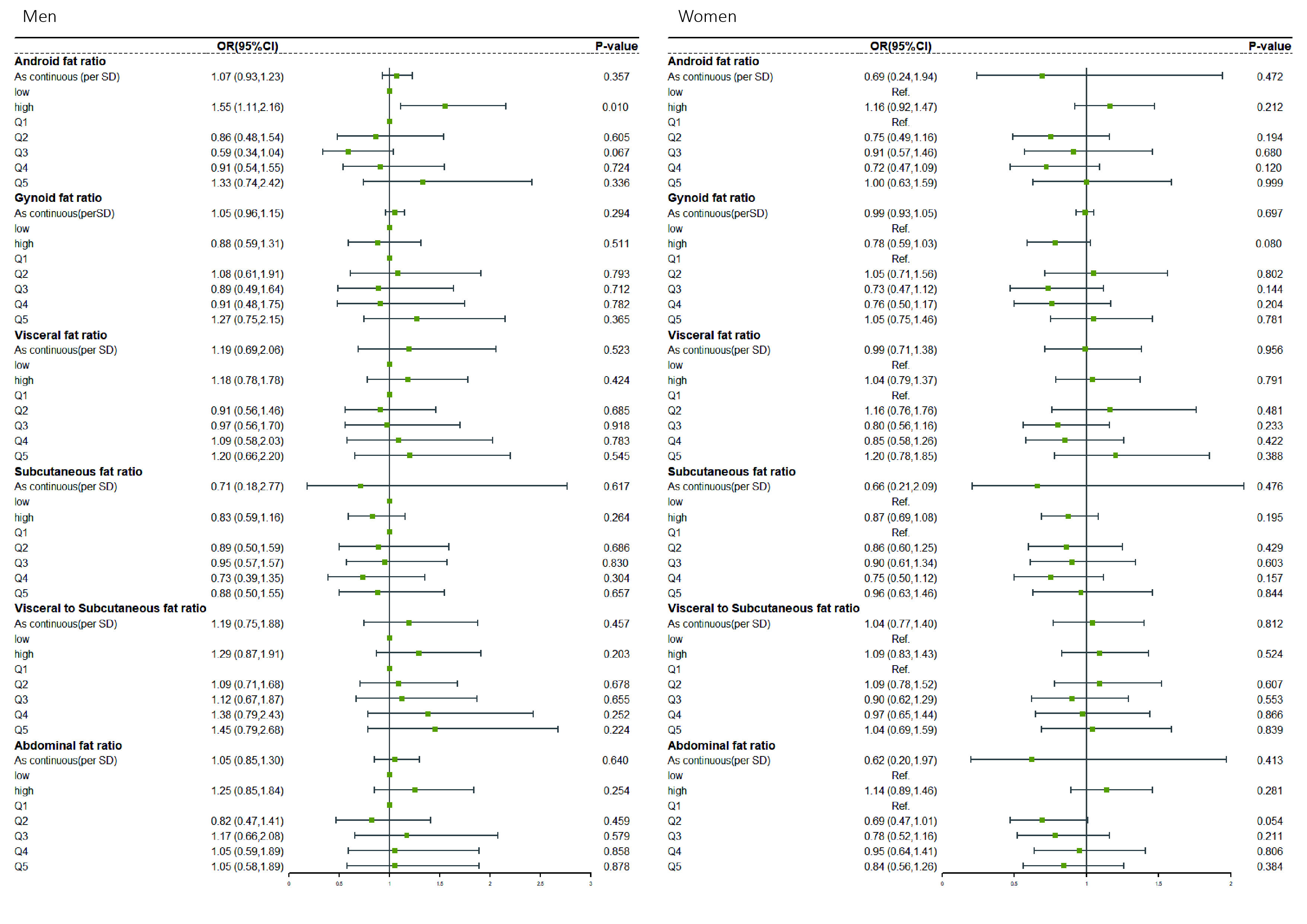


Figure S8 Relationship between different fat distribution and liver diseases in obese participants


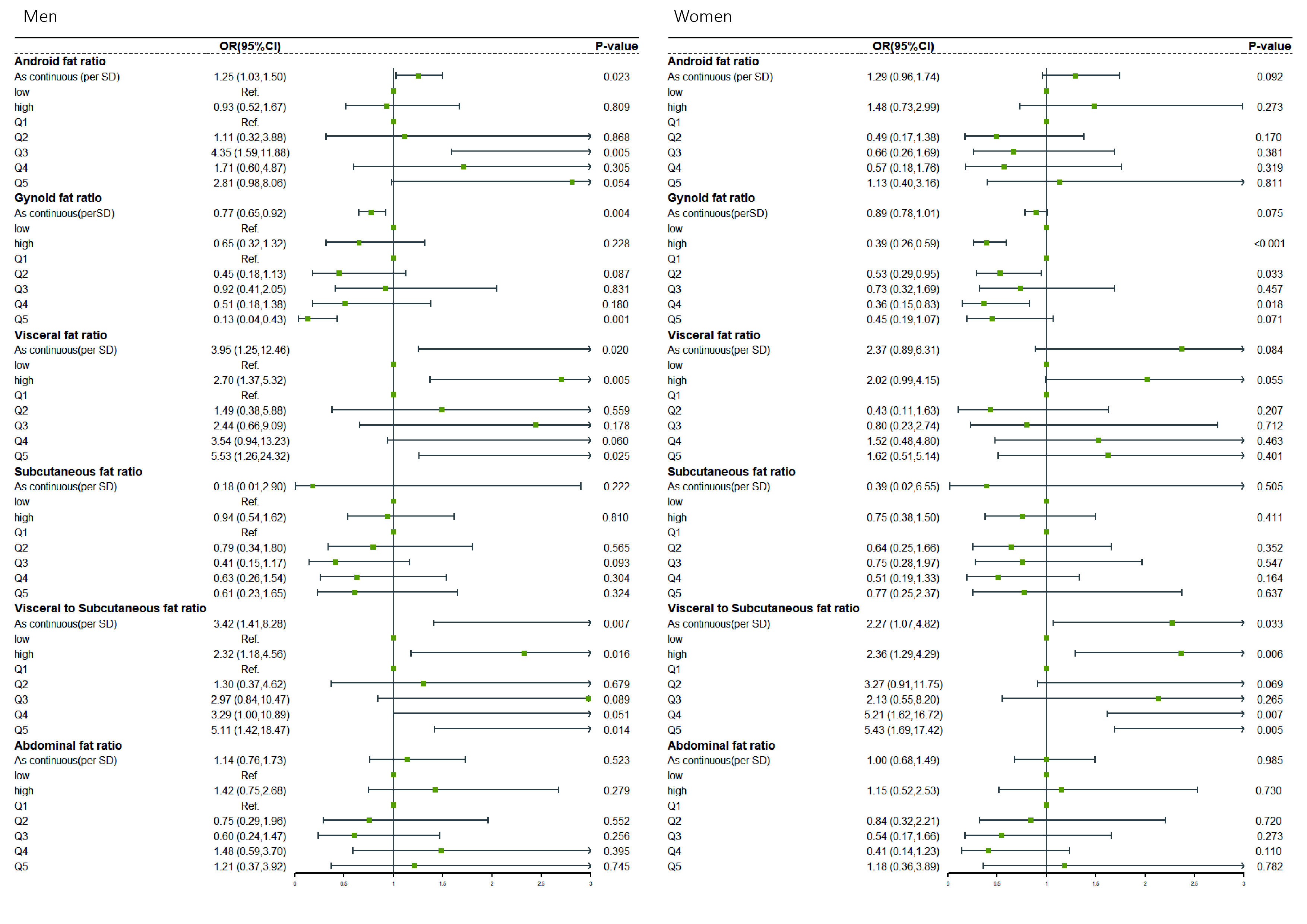


Figure S9 Relationship between different fat distribution and renal diseases in obese participants


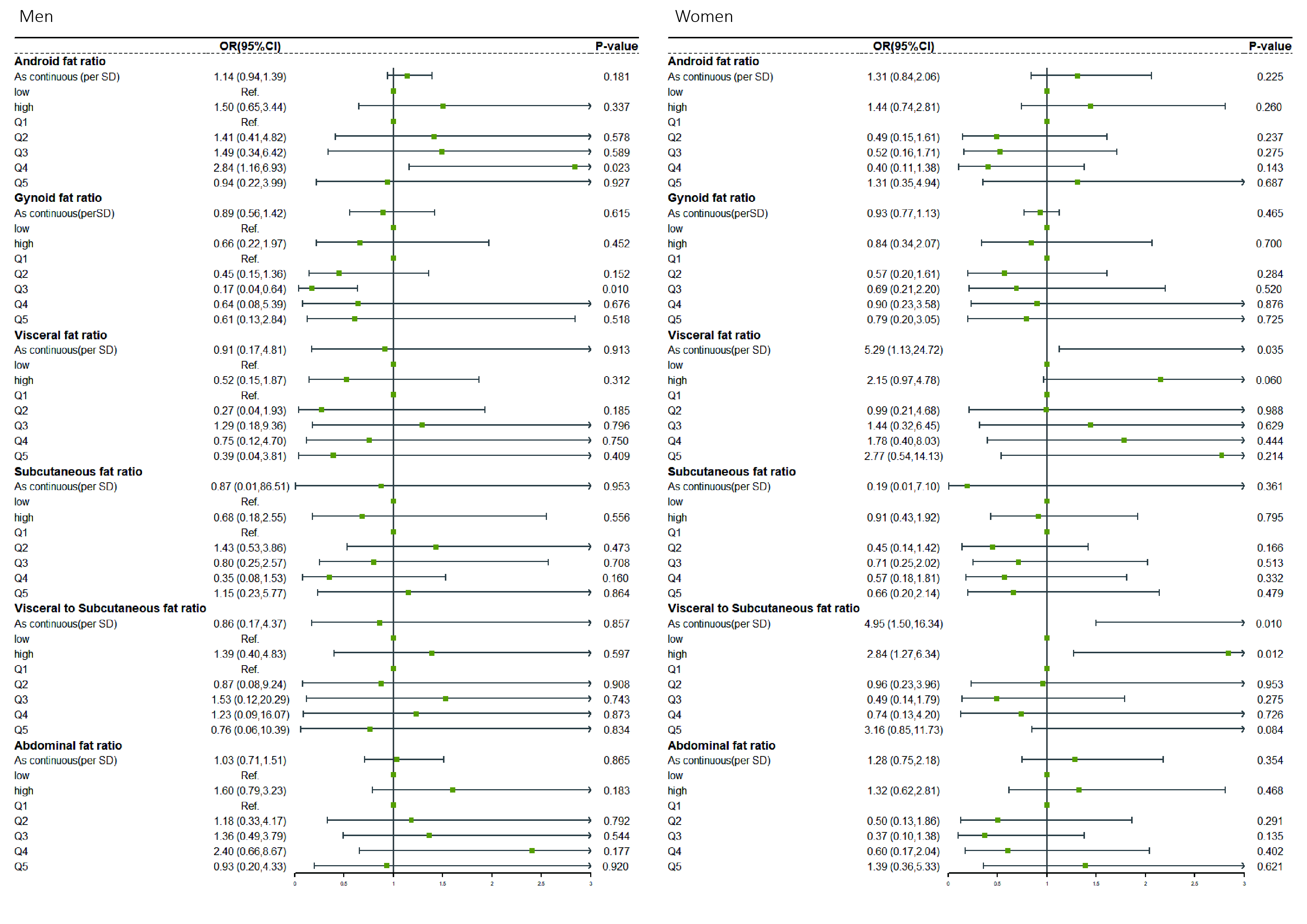


Figure S10 Relationship between different fat distribution and cancer in obese participants


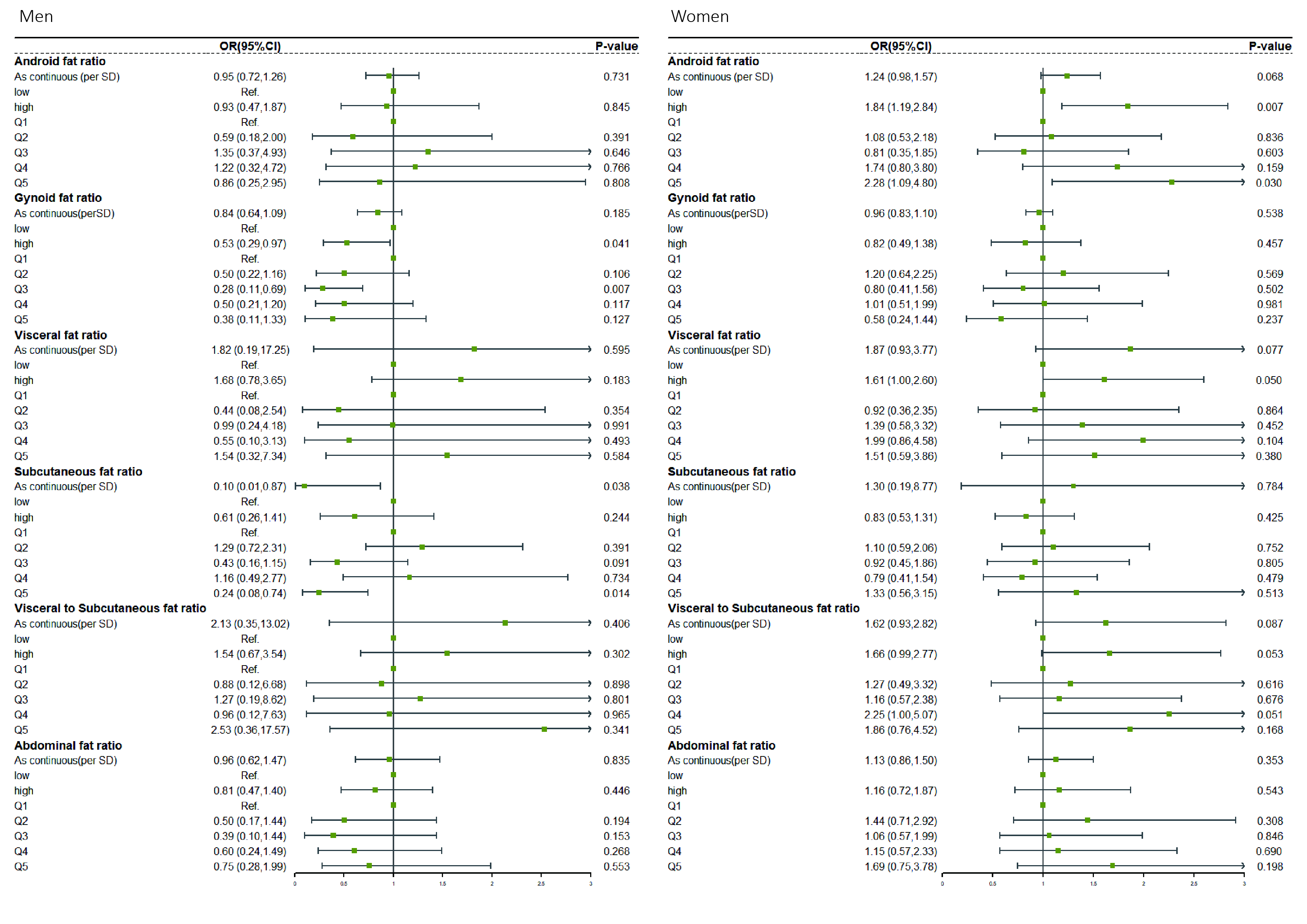


Figure S11 Relationship between different fat distribution and arthritis in obese participants


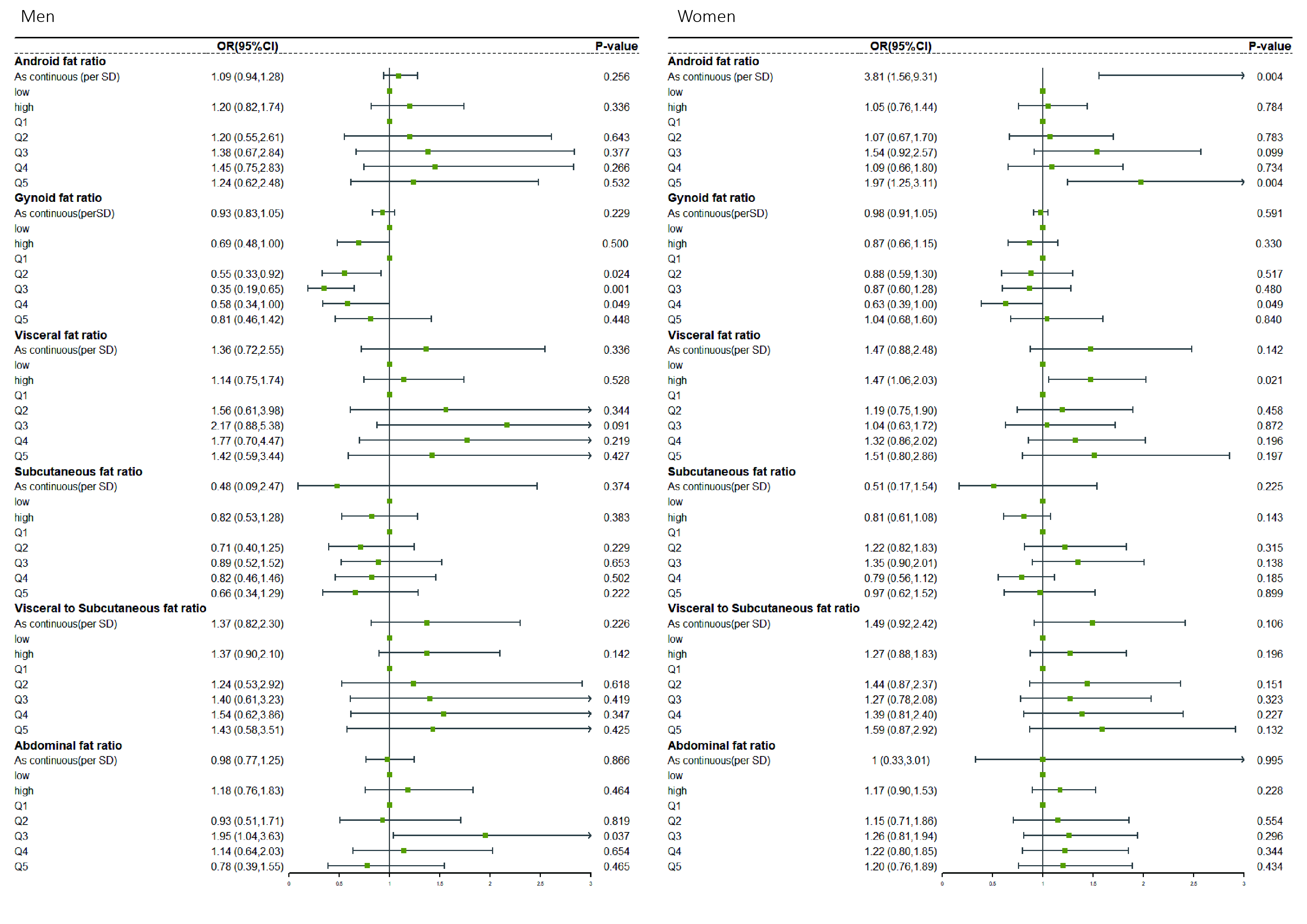


Figure S12 Comorbidity risk odds ratio (95%CI) of different fat distribution in obese men


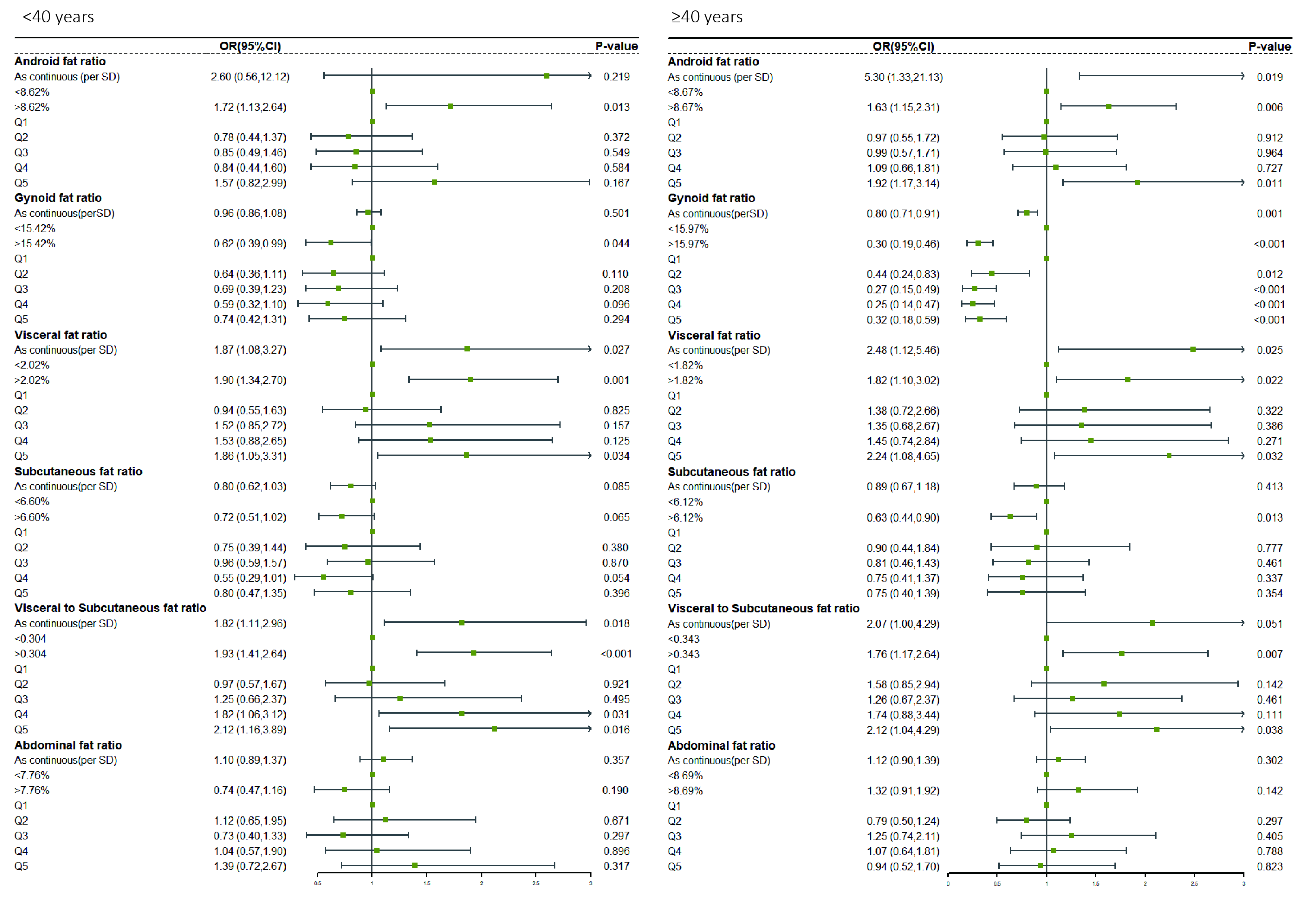


Figure S13 Comorbidity risk odds ratio (95%CI) of different fat distribution in obese women


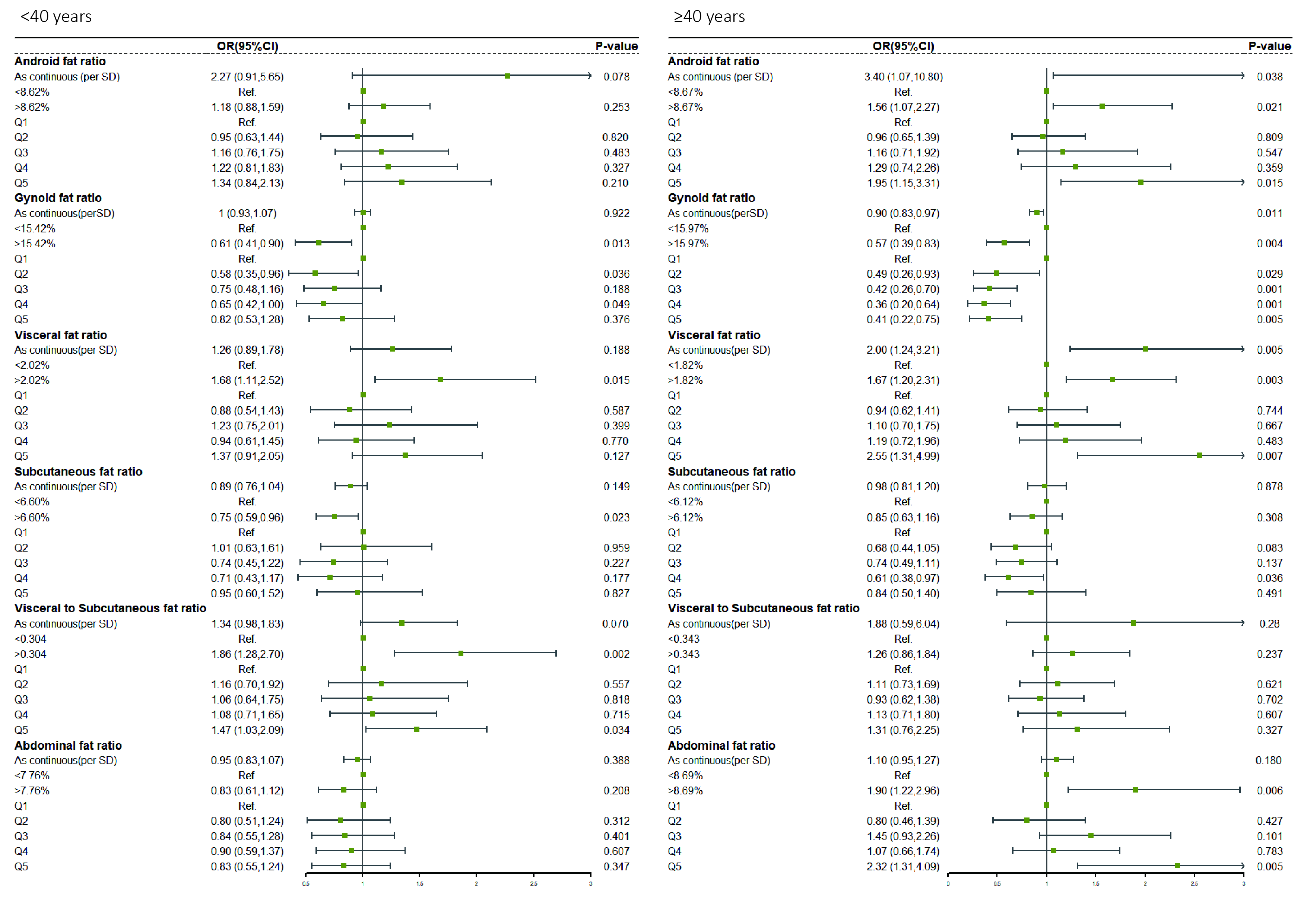


Figure S14 Comorbidity risk odds ratio (95%CI) of different fat distribution in obese women with different menstrual status


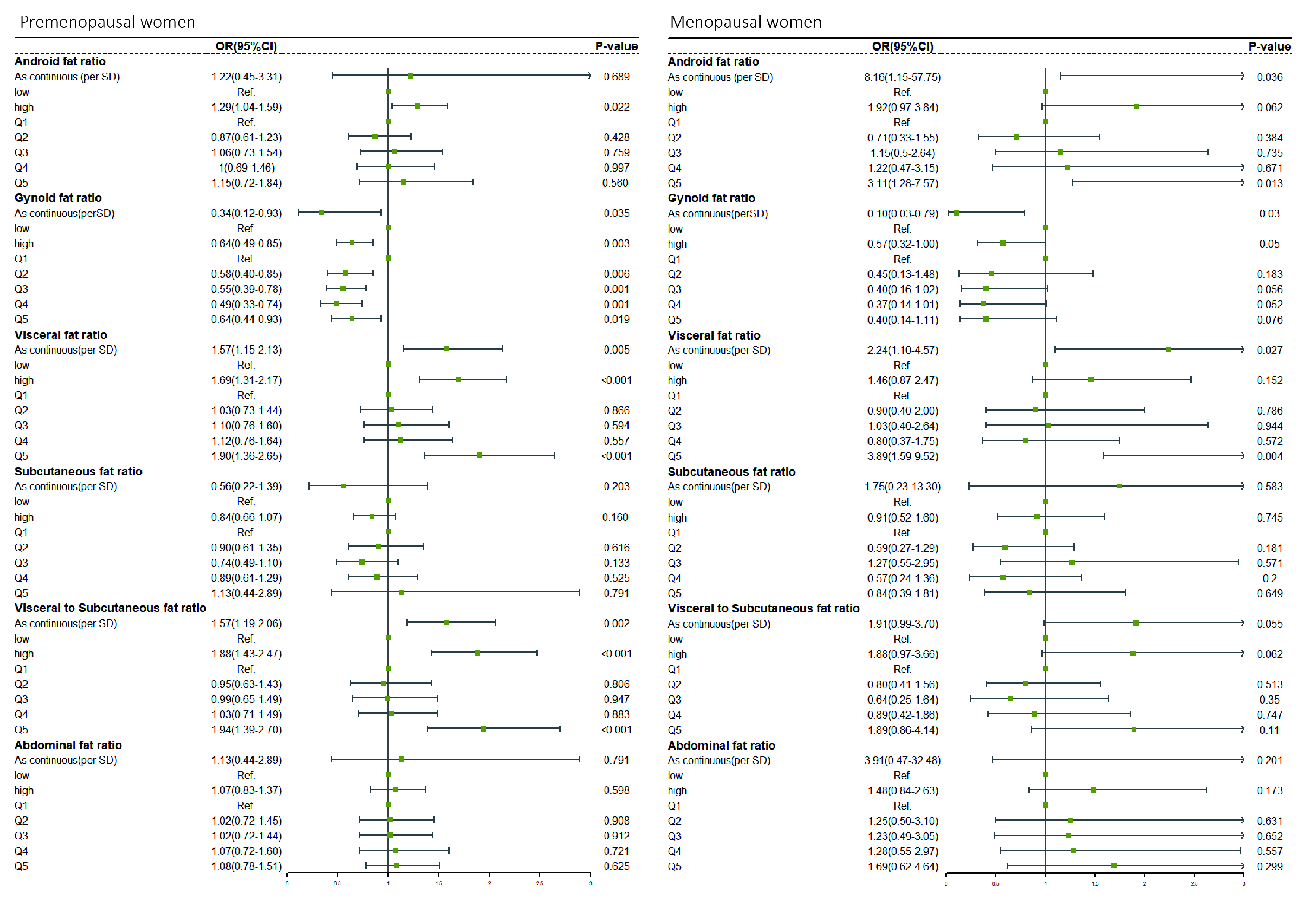

Supplement: Supplementary file 1 [file DataSheet_1.docx]
